# Selections from the Foreign Periodicals

**Published:** 1847-10-01

**Authors:** 


### CIRCUMSPECTIVE REVIEW.

---

### Selections from the Foreign Periodicals.

---

### On the Frequency of the Pulse and Respiration of the Aged. By C. W. Pennock, M.D.

"Physiologists, generally, have considered it as an established fact that the frequency of the heart's action diminishes in advanced age; and no one has called the correctness of this view into question until Leuret and Mitivié in 1832, whilst engaged at the Salpêtriere in observations relative to the pulse of the insane, were astonished to find that the pulse of 34 sane women, in good health, whose medium age was 71 years, presented the average of 79 beats in the minute. This fact induced them to make further observations, and to institute an inquiry as to the relative frequency of the pulse of the young adult and that of the aged. On the same day, at the same hour, and under analogous circumstances, the pulses of the young men at the Veterinary School at Alfort, and those of the old men in good health at the Bicêtre were examined. The number of the veterinary students was 110, that of the aged men 27: the average age of the students was 21, that of the aged men 71 years. The result of the examination proved that the medium pulse of the young men was 65, whilst that of the aged was 73. Temperature 32 F.

"Evidence confirmatory of the view of Leuret and Mitivié was offered in 1835, by Drs. Hourman and Deschambre of Paris, who re-investigated the subject of the pulsations in connexion with that of the respiration of the aged. Their observations were made on 255 females, in good health, between the ages of 60 and 96 years of age, the average being 74·33 years. These researches were chiefly made between 6½ A.M. and 7½ A.M. at the temperature of from 46° to 48° F., soon after the individuals had left their beds and previously to eating. The result of the investigation was as follows: viz. medium age 74·33 years; medium number of pulsations 82·29; medium number of respirations 21·79. The ratio of the frequency of the respiration to that of the pulse as 1 : 3·41."

Dr. Pennock next furnishes us with an account of the results of a series of observations upon the pulse and respiration of the aged inmates of an Infirmary adjoining the Philadelphia Hospital, Blockly. They were usually made at least four hours after breakfast, when the individuals were undisturbed by exercise and free from mental excitement, the precaution being taken in order to familiarize them with the proceeding of making observations prior to those which were recorded. Various medical friends likewise forwarded to him the results of their own investigations, which he has embodied in the present paper.

"Rejecting all observations of individuals in whom any rational or physical signs of cardiac, pulmonic, or other disease existed, the number of persons whose pulse is reported is 170 men and 203 women: being an aggregate of 373: the ages of the men being between 50 and 90, those of the women between 50 and 115. The frequency of the respiration does not seem to have claimed that attention from physiologists which it merits. It has been an interesting subject of inquiry in connexion with that of the pulse, and the effort has been made to ascertain the ratio of the inspirations to the cardiac contractions."

The particulars of these observations are set forth in tables: but we must confine ourselves to the general statement of results furnished by the author.

"Table A. is derived from the observation of the pulses of 170 *men;* the aggregate of whose age was 10,895, and that of the pulsations 12,211. The respirations were counted in 146 instances, the total number of inspirations being 3045. The medium age was, therefore, 64·09 years: the medium pulse 71·83: the medium respiration 20·51; and the ratio of respiration to pulsation as 1 : 3·51.

"Table B. is derived from the observations of the pulse of 203 females, the aggregate of whose ages was 14,326, that of their pulses 15,838. The respiration was counted in 143 individuals, and its aggregate was 3154. The medium age was 70·57 years; the medium pulse 78·02; the medium respiration 22·06; and the ratio of respiration to pulse 1 : 3·53.

"From the preceding facts it would seem to follow that, the medium pulse of the aged men may be stated to be 71·83; that of aged females 78·02. Whilst the respiration of the former is 20·51, that of the latter 22·06 per minute. The ratio of the respiration to the pulse in aged men is as 1 : 3·51, in women as 1 : 3·53.

"The idea may be entertained that the frequency of the pulse of the aged, as stated in the preceding tables, may depend upon some accidental instances of extreme frequency, which should be rejected. In order to the proper appreciation of the truth, the tables C and D are presented, in which the individuals examined are grouped agreeably to the frequency of the pulse. From the table C it is very apparent that, in above one half of the aged men, 52 per cent., the pulse ranges from 76 to 84; that in more than one-third, 43 per cent., it is over 63; whilst in about 2 per cent. it averages, say 55, and in rather more than 3 per cent. it is over 96. From the table D. it is evident that the pulse of aged females varies from 70 to 104 in nearly four-fifths of the individuals—79·93 per cent. of those examined; that in more than two-thirds, 69·45 per cent. the range of the pulse was between 75 and 86; that in 7·39 per cent., the pulse was between 95 and 96; that in 2·41 per cent. it was at 104; whilst it was below 70 in but a small number, namely, 37 out of 203, being rather less than a fifth of the whole, or 18·34 per cent. The pulse was below 60 only in 5 instances, or in 2·41 per cent. of the whole number."

After comparing these results with those obtained by the above-cited Parisian enquirers, Dr. Pennock thus concludes.

"From the preceding facts and researches, it is evident that the frequency of the pulse of the aged is much greater than that usually assigned to it; whilst that of the respiration is equal to that generally admitted in reference to the adult in middle age. 'Generally,' say Hourmann and Deschambre, 'it is in old age that the pulse presents extremes of slowness or of frequency; but the first case is the exception, the second is the rule: the error of past time has been to take the one for the other."—*American Journal Med. Sciences,* July 1847.

[Every attentive practitioner must have had occasion to verify the truth of the above proposition respecting the pulse, though probably on a smaller scale and less exactly. It is a point of great importance to be borne in mind, not only while directing the treatment of a case, but in prognosticating the soundness of a convalescence, which the persistence of a supposed anormal frequency of pulse might otherwise seem to forbid.—*Rev.*]

---

### On Scorbutus as observed in the Salpetriere in 1847, and on the Composition of the Blood in this Disease. By Dr. Fauvel.

Since Scorbutus, yielding to the progress of hygiene, has almost disappeared in localities wherein it was heretofore endemic, no important work has appeared upon the history of this disease, of which the physicians of the last century have described the frequency and terrible effects. The work of Lind, so replete with facts and erudition, has not been surpassed, and the picture he sketched of the symptoms of the disease has served for the model of the most recent descriptions. The researches upon the composition of the blood in various diseases, undertaken with so much *éclat* in our own times, has however recalled the attention of physicians to this malady of a prior epoch. The reason of this new interest may be readily understood, when we consider that the fate of an entire doctrine depended upon the solution of the problem of the condition of the blood in Scorbutus.

Chemistry having confirmed the importance of the inflammatory crust of the blood which clinical experience had long since ascertained, it was next inquired, whether, in another pathological condition, an inverse appearance of the blood did not correspond to an inverse chemical change. This question was only imperfectly answered by the analysis of the blood in some of the pyrexiæ, since in these a diminution of the fibrine was found to be far from manifesting itself as a constant fact in these diseases. Was it otherwise in regard to Scorbutus? Everything led to the supposition. The dissolved state of the blood, mentioned and differently interpreted by Hoffman, Boerhaave, Huxham, Lind, and many others, gave a great probability to the conjecture: while Magendie succeeded in inducing phenomena in animals, analogous to those of Scorbutus, by the injection into the veins of defibrinated blood or alkaline solutions.

Such was the state of the question when, in 1841, Andral, analysing the blood of a man affected with scorbutus, found the proportion of fibrine less than the normal mean; and from that time the former conjectures were considered as confirmed; a rational explanation of symptoms was given; and an ingenious theory of hæmorrhages, which appear in certain diseases of which scorbutus may be considered the type, was furnished. In the phlegmasiæ in which there is an increased quantity of fibrine in the blood, no hæmorrhagic tendency is observed: but in typhus, scorbutus, and in other diseases in which the blood is in a more or less dissolved state, that is containing little fibrine, hæmorrhages are frequent, and are produced independently of the essential differences which separate the various diseases. The hæmorrhage is then a common phenomenon, dependent upon a common change in the composition of the blood in all these cases. If to this we add, that in certain other morbid states, in which the quantity of globules is greater than in the normal state, at the same time that the fibrine is unchanged, the hæmorrhagic tendency is produced but with different characters, we naturally arrive at the distinction founded upon a change in the composition of the blood peculiar to each of these.

In vain did Mr. Busk declare that in several scorbutic patients he had found the fibrine augmented; and in vain did M. Stæber refer to the conclusions of Parmentier and Deyeux that the scorbutic blood they examined was identical with inflammatory blood. These statements were received with incredulity, as arising from some error in diagnosis or the process of examination. Yet it was desirable that new and numerous analyses should sanction the principle laid down by M. Andral from a solitary case: and this year an unusual opportunity offered itself to me at the Salpêtrière. Among the old women there, a few spots of ecchymosis on the limbs are not infrequently seen: but for more than a year no case of scorbutus had shewn itself; when last March, a woman æt. 76, strong and well for her age, became suddenly attacked by symptoms of it. Certain of these symptoms led to her being bled, and as no inflammatory complication was present, the usual state of the blood described by authors was expected to be found; but to my astonishment next day I found the clot covered by a yellow, firm, elastic, fibrinous layer, just such a one as is met with in simple pneumonia—the remainder of the clot offering no signs of dissolving. MM. Becquerel and Rodier were made acquainted with this unexpected result, and as a little epidemic of about thirty cases manifested itself during the next two months, they had ample opportunity of instituting more exact enquiries into the condition of the blood. The blood of five patients were submitted by them to analysis, and the following are the conclusions arrived at by these able chemists.

"1. That far from presenting a dissolved state, as the usually received theory supposes, the blood in these cases offered a well separated, and sometimes very firm clot, swimming in a limpid serum uncoloured by the presence of globules. 2. The density of the defibrinated blood was in all the cases below the normal mean (1057), and the figure 1038·3 observed in one case was lower than had ever yet been met with by MM. B. and R. Moreover this diminution of density, in the five cases, was not in relation with the respective proportions of water and solid matters of the blood—an extraordinary fact quite inexplicable. 3. That the density of the serum was notably lower than the normal figure. 4. That the quantity of globules was always less than normal, and was found very much diminished in the case corresponding to the lowest density of the blood. 5. That the *fibrine*, contrary to received ideas, *was not diminished in any of the cases.* In two cases it offered the normal proportions (2·2), while in the three others it was sensibly augmented, viz. 3, 3·6, and 4·1. It appeared to be endowed with all its ordinary physiological properties. 6. That the organic matters of the serum, of which *albumen* constitutes the larger portion, had undergone a notable diminution, while the water existed in a considerable proportion. 7. That in no case were the inorganic matters superabundant, and nothing proved an excess of alkalis, which sometimes authors have declared to be the case in the blood of the scorbutic."

Is it not remarkable thus to see the most rational previsions entirely overturned? Theory declared that in scorbutus the quantity of fibrine was diminished, whence arose a defective plasticity, the cause of the characteristic hæmorrhages. It added that such diminution of fibrine might be the sole change, without necessitating that of the other organic elements of the blood. But the exact reverse of this has come to pass. The *globules* and *albumen*, which should have remained uninfluenced, are found to descend much below the physiological mean; while the *fibrine* exhibits an opposite tendency, so as to approach it to the condition observed in inflammations. Since the above analyses have been made, M. Andral has himself made another, and found the fibrine as high as 4·5; and M. Prus states that, he has frequently observed, at the Salpêtrière, facts analogous to these now brought forward. On the other hand, is it not interesting to see so large a diminution of *albumen* without any dropsy resulting, save a little œdema in the neighbourhood of much ecchymosis? Is there not here something which materially interests the theory of the production of dropsy by a diminution of the albumen of the blood?

But it will be said, do you pretend by these few cases to invalidate all the descriptions of the state of the blood in scorbutus handed down to us by the old writers? By no means. Only we would draw different conclusions from them. In fact, if we consult the principal authors who have written upon scorbutus, it becomes evident that they have given this name to diseases of a different nature, to true typhus; or that they have not taken into account the intercurrent affections arising during an epidemic of scorbutus, as of those which result from a putrid infection produced by the crowding together of patients in too small a space, as on board a ship. The dissolved state of the blood occurring under these circumstances would be easily explained.

And even supposing that in the great and destructive epidemics of scorbutus, the blood, deprived of fibrine, lost its power of coagulation, does it follow that this should always be the case? Is the quantity of fibrine constantly diminished in typhoid fever? To arrive at a knowledge of a disease, is it not necessary to study it in its most simple form, and to disengage it as much as possible from the complications which change its essential characters? It is precisely because the scorbutus we have observed presented itself in a simple but characteristic form, that the results we have adduced have an undeniable importance. They are neither numerous or conclusive enough to serve as a basis for any particular doctrine. They have only a negative value as tending to cast doubt on the doctrine of scorbutus prevailing in our time. They demonstrate the necessity of new researches, directed in other manners and by other proceedings, and undertaken upon a vast scale. Lastly, they confirm the utility of bearing in mind the maxim which Lind called the attention of the physicians of his time to—*Chymia egregia ancilla medicinæ; non alia pejor domina.*

Dr. Fauvel supplies a minute medical history of the cases observed by him, in proof of their being examples of genuine scorbutus: but to this we need very briefly refer. It showed itself chiefly in persons advanced in age, the youngest being 69 and three more than 80. They were all previously in good health, and fed upon substantial food. Among the principal symptoms observed was—(1). A *yellowish colouration of the entire skin, quite sui generis,* most comparable to the light yellow tinge which succeeds to ecchymosis. It affected especially the face, and extended to the conjunctivæ; but the urine giving no traces of colouring matter proved it was not icterus, which it so much resembled. This colour diminished and disappeared in proportion as the case proceeded on to recovery. (2). The *hæmorrhagic spots* offered very different characters even upon the same subject, varying from mere red points to true purpural petechiæ. The most important variety was a true scorbutic hæmorrhage, consisting of large ecchymoses or infiltrations into or below the subcutaneous cellular tissue of the lower extremities—occasionally constituting quite projecting tumefactions. Great pains frequently accompanied these effusions; and indurated œdematous infiltrations of the limb, together with pain and tenderness of the joints, were sometimes found. (3). The *changes of the gums* were quite peculiar, not consisting in a general tumefaction and softening of their tissue as seen in some stomatites; but of fungous vegetations developed around the neck of each tooth, and being therefore only numerous in proportion to the number of teeth which remained. They were soft, and bled easily. Mastication was difficult, and the mouth exhaled a very fetid odour. (4). The *prostration of strength* of both body and mind was remarkable; although the tendency to syncope, mentioned by Lind, was not remarked—the patients, however, retaining the recumbent posture.

The *treatment* employed consisted in the use of a tisane acidulated with lemon, a mixture containing tannin and antiscorbutic syrup, linseed cataplasms watered with a decoction of bark, as substantial a regimen as the state of the mastication admitted of, composed of rich soups, roast meat, and as large a proportion of green vegetables as possible. In the course of treatment the juice of antiscorbutic herbs was substituted with great advantage for the tannin. Of nine patients, four were completely cured in an average period of two months, four still continued under treatment, and one died.—*Archives Generales,* t. xiv., pp. 262-287.

Subsequently to the results of MM. Becquerel and Rodier's investigations being communicated to the Academy, *M. Andral* read an account of a new analysis which he had at the same time made of the blood drawn from a well-marked case of scorbutus under his own care at La Charité. The following is the substance of his important remarks.

" I expected to find it diffluent and dissolved, but to my great astonishment it was not so. It consisted, in fact, of a small dense coagulum as resistent as that of phlegmasiæ, and covered by a well-marked buffy coat, and was suspended in a large quantity of serum. In the 1000 parts it gave the following proportions:

| | |
|---|---|
| Fibrine.. .. .. .. .. .. | 4·420 |
| Globules .. .. .. .. .. | 44·400 |
| Solid matters of the Serum, | 76·554 |
| Water.. .. .. .. .. .. | 874·626 |
| | 1000·000 |

"This blood in its composition resembled that of chlorotic patients by the diminution of its globules, and the large quantity of water, although the patient presented other symptoms than those of mere chlorosis. As to the fibrine, instead of being less than, it exceeded, the physiological mean. This is quite a different result from that I obtained in the other published case, in which the globules were in nearly their normal proportion, and the fibrine was in very small quantity. So, too, I found very little (about 1 per 1000) in a patient attacked with purpura hæmorrhagica.

"The fact which I have narrated, and which is confirmatory of those recently laid before the Academy, proves that the symptoms ordinarily characterising scorbutus may be produced without being necessarily accompanied by a diminution of fibrine. It is not then in such diminution we can place the proximate cause of scorbutus; and it is not by it even we can hope to explain several of its symptoms, especially its characteristic hæmorrhages. In this respect, as well as in several others, we may perhaps compare scorbutus with *typhoid fever*. In the latter, in fact, the lowering the proportion of fibrine is often met with, but it is not necessary for the existence of the disease. Observation only authorizes us to state that the diminution is considerable in proportion as the dynamic form of the disease is well pronounced. The same thing takes place in the eruptive fevers; so that, in these different cases, the diminution of fibrine cannot be considered as one of the necessary elements of the disease, but only as one of the more or less frequently occurring effects of the cause which has produced it, and which exerts its influence alike upon the vital forces which it tends to depress, upon the nervous system which it throws into great perturbation, and upon the blood in which it tends to produce an alteration, the reverse of that observed in phlegmasiæ. The same thing appears to me to take place in scorbutus. Like typhoid fever, it may become developed without the blood having previously lost its fibrine, and therefore a diminution of this is neither a necessary and constant occurrence, but only an effect, a result of prior morbid modifications, a result which is produced more or less frequently according to the severity, duration, &c., of the disease.

"Are we to refer the hæmorrhages of scorbutus to this diminution of fibrine as their cause? There can be no doubt that hæmorrhages which, like those of scorbutus, are multiplied and repeated at a great many points of the economy, are especially seen in those cases in which the blood itself has become more poor in fibrine than in the physiological state. But these are two facts which it seems to me are only coincident in their relation: both doubtless being most frequently produced under the influence of a common cause: both are but manifestations of that cause, but the one does not seem to me to engender the other. The case I have related presents an example of remarkable hæmorrhages without any such diminution. Moreover, is it so easy to understand theoretically why a diminution of the quantity of fibrine should lead to a flow of this fluid from its vessels? Is the diameter of the globules diminished, so that they can more easily traverse the vascular parietes? Are we to admit that this passage may be favoured by an antecedent hypnæmia, when, after the violent distension of the vessels produced by inflammatory congestion such effect is not produced, and the globules do not leave their vessels but when these have been torn? This opinion, reproduced in our times, and which I myself have maintained, making the hæmorrhages of scorbutus and pernicious fevers dependent upon a dissolved state of the blood, arose at a period when it was believed that this took place from the destruction of its globules; but such destruction, supposed by Huxham and so many others, is a pure hypothesis. Many a time, in cases in which the blood presented different degrees of this dissolved state, have I examined it with the microscope; and I have constantly found its globules with their ordinary appearance, and perfectly untouched. So, too, the globules in the blood from the hæmorrhages possess all their physiological characters. I repeat, then, it is not easy to understand how the simple diminution of the fibrine should render their issue from the vessels more easy. In considering the augmented portion of fibrine observed in this patient, we must bear in mind, however, that when the bleeding was practised there were symptoms of acute disease of the respiratory organs, with much febrile re-action, and I believe it is to this circumstance we ought to attribute so large a proportion of fibrine. Some days after this bleeding had been practised, and probably in consequence of it, we could discover no traces of inflammatory action. Although the blood, at least three weeks prior to death, had not lost its fibrine, the spleen, nevertheless, was none the less soft and reduced to a pulp; so that we here see a change which, although generally coinciding with a dissolved state of the blood, may yet exist without it. The highly congested lungs, containing here and there apoplectic kernels, contrasted by their dark colour with the extreme paleness of the liver and kidneys."—*L'Union Medicale,* No. 78.

M. *Marchal* (*De Calvi*) in a communication to the Academy of Sciences, expressed his doubts of the correctness of the conclusions recently arrived at, from the fact of fibrine being sometimes found in excess in the blood in scorbutus. He believes the inflammatory element, to which this is due, has been overlooked. He lays down the following propositions:—1. There are two species of hæmorrhagic phenomena in scorbutus; sanguineous infiltration or interstitial hæmorrhage, and hæmorrhage properly so called. 2. Each of these may be external or internal. 3. In internal hæmorrhage the infiltrated parts may re-act and inflame. 4. It is such re-action that explains the maintenance of the normal or production of the excess of fibrine. 5. In hæmorrhage properly so called it is to be presumed that, as there is no local re-action, the amount of fibrine will continue less. 6. There is no reason, from our present information, to change our opinion of the defibrinated condition of the blood in scorbutus. 7. Even were this the case, it would offer no reason to deny the defibrination of the blood in the pyrexiæ, and the dependence of the hæmorrhagic phenomena upon this circumstance. 8. In the present epidemic, besides the changes introduced into the regimen, another indetermined cause has acted. 9. The albumen and globules are diminished in scorbutus, and yet, in general, there is neither dropsy or arterial murmurs. 10. As to the absence of dropsy, we may explain it easily, the *deficiency in the formation* of albumen being a very different thing from the *loss* of this principle. 11. Scorbutus and Typhus are not analogous. In the one there is impoverishment, in the other, a poisoning of the blood.—*Gazette Medicale,* No. 34.

In relation to one point, alluded to in M. Andral's communication, the *analogy prevailing between Scorbutus and Typhoid Fever,* we may notice a short discussion which recently took place at the Academy of Medicine.

An epidemic of Scorbutus having broken out in the garrison at Givet, M. *Scoutteten* was sent by the Government to investigate its causes. He considered that the causes of the disease were similar to those capable of inducing typhoid, the hospital being placed in a very unhealthy locality, and the regiments whence the patients were derived having suffered under a defective alimentation. The patients were removed from the damp locality in which they were placed, and soon did well upon a good diet. The particulars of the diet the soldiers had been subjected to he was not at liberty to divulge. (This disgraceful fact has been well and severely commented upon by the entire Parisian medical press). M. *Rochoux* having stated his opinion, that a vitiated state of the air had always been the leading cause of the production of this disease, M. Scoutteten felt certain that, however much other causes might aid, defective alimentation was the principal one. Thus, when the new prison regimen, which included a diminished diet, was adopted, scorbutus quickly showed itself; and as quickly disappeared when the diet was improved, all other circumstances remaining the same. In reference to the present epidemics, two regiments occupied the same barracks, one being much better fed than the other, and the latter alone suffered from the disease. M. *Ferrus* observed that scorbutus much prevailed in prisons and lunatic asylums at the present time; and that, although bad diet was the principal, it was not the only cause. He had found it very often co-existent with other diseases, especially typhoid, owning common causes. "All these affections seem connected by one common tie—general debility, and are we to attribute this to humidity, alimentation, or over-crowding? To all of them united. We find in such places an assemblage of a great number of individuals in a too circumscribed space, an insufficient and too uniform a diet, a sedentary life for persons mostly accustomed to laborious occupations, and the demoralisation and despondency contracted by their situation." M. *Bouillaud.* "We have heard nothing but assertions unsupported by facts. It is quite evident to those who have meditated upon the causes of scorbutus and typhoid, that not only is there no identity, but not even any analogy. Daily may we see, in the hospitals of Paris, cases of typhoid fever, and for my part I have seen more than 700 in 25 years, and yet I have never seen a single case of scorbutus. If the analogy is so striking, why do not the same causes produce both effects? Some persons admit that typhoid fever is contagious, but no one will allow that scorbutus is so. In typhoid there is febrile reaction, none in scorbutus." M. *Scoutetten.* "I am much surprised to hear that M. Bouillaud has never seen scorbutus. He has only to visit the wards of Val de Grâce, where an epidemic now prevails; and he may there see typhoid fever and scorbutus developed in soldiers subjected to the same influences."—*Gazette Medicale,* No. 29.

[The subject of Scorbutus has excited so much interest during the last few months in various parts of Great Britain—practitioners who had began to consider it as a mere traditional disease, having now had ample opportunities of studying its various features—that we have deemed it right to lay the above somewhat lengthy extracts before our readers. We cannot consider that the analyses here referred to definitively settle the question of the amount of fibrine ordidarily contained in the blood in this disease, and are disposed to adopt M. Marchal's suggestion. Our means of investigating the condition of this fluid are far superior to those possessed by the older observers; but they were as well able to estimate the mere appearance of the coagulum, as influenced by the presence of more or less fibrine, as ourselves. As to the causes of the disease, these, in common with those of other epidemic visitations, are enveloped in the obscurity implied in the phrase "epidemic constitution of the air;" for, although a defective or ill-adjusted alimentation seems essential to the production of the malady, it does not *alone* suffice, as, did it so, the disease would have been found still more generally prevalent during the late season of distress and privation, and would have also frequently manifested itself on former similar occasions, instead of being a new affection for almost the whole race of living practitioners.—*Rev.*]

### Influence of Electricity in the Production of Diseases. By M. Pallas, Principal Physician in Algeria.

Dr. Pallas has addressed a note to the Academy of Medicine prior to publishing a work he has undertaken upon the importance of electrical isolation in the treatment of certain diseases. The subject, he states, is especially important in relation to the etiology, nature, and treatment of the diseases of hot climates; and may be summarily stated in the following propositions.

1. The greater number of diseases, and especially those which belong to the class of neuroses, are occasioned by the exaggerated influence of general electricity, of which clouds, storms, and marshy regions are the most fruitful sources. 2. Marshes, in their geographical constitution, and the effects which they produce upon the economy, present the greatest analogy with the galvanic pile. Thus their action is so much the more baneful as they contain certain proportions of water, and their activity is considerably increased when the water contains organic or saline matters in a state of solution. This explains why salt marshes and such as are near maritime rivers are the most insalubrious. The drying up or submersion of marshes produces analogous conditions to those of a galvanic pile deprived of humidity, or which is under water, and the effects of which are then insignificant. 3. The researches of philosophers and physiologists have shown that the electricity produced by our machines exerts a special action upon the nervous system. Experience and rigorous observation of facts prove that the diseases which are produced in a marshy atmosphere are primarily nervous, and become inflammatory only by the re-action of the nervous upon the vascular system, inducing consecutive local or general irritation. 4. The neuroses are occasioned, generally, by the effects of electricity, and intermittent fevers have a similar origin, that is to say, they are due to the electrical emanations of the marshy pile, which are very active in hot countries, and not to miasmata, which have never been met with. 5. Electrical isolation is a rational means of modifying this morbid influence, and is accomplished by attaching to ordinary beds, sofas, or chairs, legs of glass or resin. A great number of cases prove that the patients whom I have thus isolated, have been cured or relieved, several of whom had resisted all the ordinary means of cure.

" 6. Just as light and air are the essential agents of vision and respiration, electricity is the functional agent of innervation, whose injurious action may be modified by isolation, which is to electricity what a shadow is to the solar light." —*Bulletin de l'Academie,* Tom. xii., p. 743.

---

### On the Diagnostic Signs furnished by Inspection of the Vulva and the Entrance of the Vagina. By M. Huguier.

By the aid of this simple means we have often been enabled to recognize certain facts, and diagnosticate some of the diseases of the uterus, the nature of which were sometimes misunderstood, or could only be ascertained by long and repeated examinations, insufficient to the end, or even inapplicable in some cases. We meet with women who, whether from fear, excess of sensibility, or real or pretended modesty, will not allow of the finger, or any instrument whatever being passed within the sexual organs; while in virgins, and in women suffering from severe inflammation of the genital organs, or from original or acquired vicious conformation, it becomes impossible to carry our means of exploration beyond the vulvar orifice. These circumstances may often render the vulvo-perineal inspection a valuable aid to diagnosis in the cure of certain uterine diseases.

And it is so in certain physiological conditions bordering upon morbid states of the womb.

*Inspection during Pregnancy.*—The reddish-violet colour of the vulva in pregnancy has been very insufficiently indicated by authors. It is quite characteristic but is not uniform. Little apparent upon the internal surface of the labia, it becomes very sensible upon the inner surface of the nymphæ, near the meatus and clitoris, and upon the tubercle anterior to the vagina—wherever, in fact, the mucous membrane becomes more delicate, thin, and transparent. This colour cannot be confounded with that which is caused by the varicose state of the veins of the genital organs in some women, and which is inseparable from the course of the venous trunks whose form and direction it reproduces. The coloration of pregnancy is diffused, and differs in its shade. It is first seen at the end of the second month, and becomes very evident during the third, so that it precedes all other sensible signs of pregnancy. Moreover, we have never seen this state in persons who did not prove to be pregnant—a circumstance of importance in a medico-legal point of view.

Another peculiarity in the vulva of pregnant women is its *humidity,* which may amonnt to even a discharge. Its cellular tissue also becomes the seat of a slight *serous infiltration.* The *piliferous follicles,* moreover, become hypertrophied, and assume on the external surface of the labia a nipple-like, granular appearance. The sudorific apparatus and the sebaceous glands also secrete more actively, from whence results an anormal secretion, and a gluey, sticky condition of parts. On the other hand, in cases which only simulate pregnancy, the same parts manifest a withered, wasted appearance.

2. *After Delivery.*—In a female newly delivered, we find an anormal dilatation of the vulvar orifice, a depression and a kind of shortening of the perineum, an enlargement of the *fourchette,* with or without lacerations, or recent cicatrices; the vulvar folds, and the nymphæ are a little fuller than usual, and a dirty, earthy reddish fluid flows out, having its specific odour. The vulvar lacerations will be found especially at the back part of the vulva, at the fourchette, and next especially at the left side, and least frequently near the union of the nymphæ with the labia, especially on the left side. They are usually triangular in form, the apex looking towards the centre of the vulvar orifice, and the base towards the thighs; which is not the manner in which solutions of continuity are disposed that result from external violence from the introduction of foreign bodies into the genital organs.

3. The aspect of the genital organs allows us likewise to indicate other particularities in certain pathological and constitutional conditions of women. In fair, flaccid, lymphatic women; in those who are chlorotic or scrofulous, or who are liable to a habitual amenorrhœa, the labia, nymphæ, and clitoris are but slightly developed; the vulva is covered with little hair, the mucous membrane is pale, shining, and cool; the papillæ and follicular prominences are but little marked; the colour of the entrance of the vulva differs but little from that of the labia and nymphæ (as I will show you upon two women suffering under uterine affection, who I will submit to your examination after lecture!); and the skin of the vulvar region is destitute of that more or less abundant pigment which we observe in dark and well-constituted women, and which contrasts very sensibly the colour of this part with that of the abdomen and thighs. It is also in these lymphatic and chlorotic women that we find the vulvar orifice and the entrance to the vagina frequently bathed with a hypersecretion of the vagina resulting from atony, or an uterine catarrh of the same nature—cases in which tonics and ferruginous preparations are indicated.

4. A few words may be added concerning the fluids which bathe the vulva. If on separating the labia we see a reddish, sanguinolent, abundant, strong-smelling fluid flow out, we may suspect the existence of a cancer, a polypus, or a fibrous body projecting into the cavity of the uterus. If the fluid is creamy, of a yellowish-white, more or less consistent, and neither clinging or elastic, it proceeds from the vagina. If there are albuminous, whitish, tenacious, transparent, hyaloidean flocculi, of a greenish-white or yellow, the liquid proceeds from the uterus, and results from a simple hypersecretion or a purulent uterine catarrh. It is to be remarked, however, that in the case of cancer, polypus, or fibrous tumour of the uterus, the folds and wrinkles of the mucous membrane at the entrance of the vulva are almost entirely effaced; the papillæ and follicles have, so to say, disappeared; the parts take on a smooth, polished and shining aspect, and a pale colour.—*Gazette des Hôpitaux,* No. 74.

[Although we imagine the modesty spoken of by M. Huguier as occasionally preventing women allowing of an examination per vaginam by the finger, would in this country certainly likewise extend to the ocular inspection of its entrance, we may as well make use of the information derivable from the proceedings of patients and practitioners among whom objections upon this ground must obviously be of rare occurrence. Some of the conclusions are of too disgusting a character, such as those relating to the habits of intercourse, &c. of the patient, to be here referred to, but others may be useful occasionally.—*Rev.*]

---

### On Stricture of the Intestine in Hernia. By Louis Chapel, of St. Malo.

An enfeebled old woman, (æt. 74), unable to give any account of its prior history, was brought into the surgical ward of the Hôtel Dieu, St. Malo, with a strangulated femoral hernia upon the right side. After the taxis had been repeatedly tried, and frictions with the belladonna ointment employed, the operation was performed. She seemed to be doing very well for some hours after the operation and had an evacuation; but she sank within 48 hours of its performance. At the *post-mortem,* slight injection of the convolutions and some recent adhesions between these and the parietes were observed; and, at one point, a portion of the intestine was observed to be extremely strictured, just as if it had been compressed by a ligature. It felt just like a fibrous ring, and its circumference was not above 27 or 28 millimetres, although that of the portion above and below was respectively 96 and 70 m. The mucous membrane near the softened part was slightly inflamed, but no other change in the rest of the canal was observed. The neck of the hernial sac was found thickened.

M. Chapel, in commenting upon this case, remarks that authors have made very few observations upon the circumstance, which he does not doubt is of more frequent occurrence than is generally believed. *Ritsch,* in 1765, presented an account of a case to the Academy of Surgery, which he believed to be unique, but in its discussion before that body, others, related by *Mertrud* and *Cutavoz,* in which marked stricture likewise existed at the point corresponding to the seat of strangulation, were referred to. *John Hunter,* in his Treatise on Inflammation, states that he has met with an occlusion of the intestinal cavity by plastic lymph, as a consequence of strangulated hernia; and *Pelletan,* in his "Clinique Chirurgicale," relates the fatal termination of an operation for strangulated hernia induced by stricture of the intestine. M. *Cruveilhier,* in his "Essay upon Pathological Anatomy," even divides such strictures into chronic and acute, and believes they may be induced by the continuous pressure of a badly applied truss. *Boyer,* treating upon the symptoms which may follow the operation, states that there are no distinctive signs proving these as resulting from a stricture or strangulation of the intestine; but that, when they do not yield to antiphlogistic treatment, it is to be presumed that they depend upon one or other of these causes. "I have observed some of these cases of stricture," says *Velpeau,* "but it seems to me that their nature has been mistaken. There is nothing permanent or organic about them. They may be dissipated in a few seconds with the fingers and a purgative usually easily triumphs over them after the operation; and it would be a piece of barbarism to stretch the organ below, as has been proposed." M. *Maisonneuve,* in his Memoir upon the "Enterotomie of the Small Intestines" (*Archives Generales,* 1845), enumerates the causes which may stricture the intestine, states that the most frequent of all is the firm constriction of the intestinal tunics at the hernial orifice. M. *Vidal,* in his "Pathologie Externe," likewise admits this cause of stricture of the intestine, observing that this is more complete and persistent in proportion as the strangulation has been of long continuance.

The case already related proves that M. Velpeau's opinions upon the subject will not always hold good, although a temporary obstruction is in all probability the general circumstance. The constriction could not have been induced here by a truss, as suggested by Cruveilhier, inasmuch as the woman had worn none; and it is most probable that it resulted from organization of plastic lymph, as stated by Hunter.

During the operation the surgeon should very carefully examine the condition of the intestine, and it is from neglecting to do this that so many see their patients die afterwards. "I perceive," says Ritsch, "in these cases, how important it is not to proceed to reduce the intestine after the division of the ring, until a portion of it has been previously drawn out, and the effect of the constriction at the seat of strangulation examined; and, if I found any obliteration of the canal which would render the passage of the fæcal matters impossible, I would take great care not to make the reduction, which would only be to devote the patient to a certain death." MM. Velpeau, Tessier, and others recommend the speedy recourse to purgatives after operations for hernia. This was warmly opposed by Sanson in 1833, who was a partisan of the physiological medicine; and certainly injections or very mild laxatives would seem to be preferable to purgatives of a drastic nature.

Supposing the surgeon has recognised the stricture, his line of conduct is still a difficult one: but the facts brought forward by Louis, in his Memoir upon the cure of hernias affected with gangrene, should lead the surgeon to endeavour to form an artificial anus. If the intestine was returned in spite of the endeavours of the operator, or if he had not remarked its condition, and symptoms of strangulation still continued, he should imitate M. Maisonneuve, who determined upon searching for the intestine two days after he had operated upon a lady 64 years of age, and whose life, by this bold procedure, he had the happiness to save.—*Revue Medico-Chirurgicale,* Tom. 1, p. 329.

---

### Researches on the Normal and Anormal Capacity of the Cavities of the Heart. By Dr. Beau.

This paper is intended by its author as an Appendix to the one on Arterial Murmurs noticed at length in our Number for October 1846. In that, he sought to establish that arterial murmurs, and especially the carotidean, are produced whenever the wave of blood, rendered too large by reason of the dilatation of the cavities of the heart, exercises too great a degree of friction upon the arterial parietes. In support of this proposition he advanced that post-mortem examinations exhibited to us dilatation of the heart as a lesion connected with those diseases which are characterised by these murmurs. On the present occasion his object is to state the confirmation of this proposition he has derived from actual *mensuration*. His mode of mensuration is a great improvement upon that usually adopted; for, instead of attempting to give the absolute capacity of the cavities, which may normally vary much, he furnishes the *comparative* capacity as derived from its comparison with that of the arterial orifices—which, from their fibrous character, are preserved nearly invariable during the changes the muscular structures of the heart are liable to. He has, however, confined himself to the comparative mensuration of the *left ventricle and aortic orifice;* since with most observers he has found the cavity of the auricle very irregular and difficult of appreciation, while the right ventricle and its auricle are found of varying capacity according to the quantity of blood that may have accumulated there during the last movements of life. Nevertheless, although exact mensuration has not been attempted of these, the right cavities were always found to possess a greater capacity than the left, and the left auricle exhibited the same variations of capacity as did the ventricle of the same side. So that, in fact, the comparative mensuration of the left ventricle and the aortic orifice supplies a statement of the capacity of the entire heart.

"I divided the heart across and perpendicularly to its axis in the middle of the space comprised between the apex of the organ and the origin of the arterial orifices. I took the diameter of the circumference formed by the ventricular walls of the left side upon the section belonging to the base of the heart, having first gently compressed it to dispel the cadaveric rigidity when this existed, and taking care to maintain the ventricular walls as rounded as possible. I then measured exactly the diameter of the cavity, without comprehending its walls, and in avoiding the *columnæ* attached to the bicuspid valves. Having taken the diameter of the ventricle, I divided the aorta at the level of the free border of the semilunar valves, and measured the diameter of the circumference of the vessel upon the section attached to the base of the heart, taking care to maintain its calibre rounded, and not including the arterial walls in the admeasurement."

The hearts measured belonged to three categories of patients. 1. Those who had died of diseases in which no arterial murmurs were heard. 2. Those who had manifested such murmurs until the period of their deaths. 3. Those in whom the arterial murmurs having existed, disappeared during the latter period of their respective diseases. The reproduction of the figures would demand more space than we have at command; and, as the real point of importance is the *comparative* size of the parts measured, the general results arrived at will suffice. In the *first* category then, in which, as far as regards the murmurs, the heart and vessels may be considered as normal, the diameter of the left ventricle equalled the aortic, or was a little larger than it. And although in different individuals the size of the ventricular diameter varied, yet, on comparing this with that of the aortic, the same relation was found to be maintained. In the *second* category, in which the murmurs were persistent, the ventricular diameter was found to be about double that of the aortic; sometimes a little more, sometimes a little less. It is on account of this relatively narrow calibre of the large vessels, that the blood entering these from the dilated ventricle exerts the degree of friction which induces the murmurs. In respect to the generation of this and some other symptoms, this *relative* dilatation of the ventricle is a matter of far higher importance than its *absolute* dilatation. In the *third* category, in which murmurs once heard disappeared prior to death, the capacity of the ventricle was found in a very slight degree larger than in the first category, and less considerably than in those of the second. This dilatation of the heart depending on its atony is not a fixed, unchangeable, condition, like that which is connected with lesion of the orifices, and it diminishes or disappears with the sounds which it gives rise to—a diminution of the volume of the pulse, and of the extent of dulness on percussion in the præcordial region, being coincident events.

Phthisis is the disease in which a disappearance of the sounds and dilatation most commonly takes place as the patient becomes exhausted by the disease. In like manner, in certain cachexiæ, as the cancerous, the same thing occurs—a permanent diminution of the mass of the blood taking place, and a corresponding gradual retractation of the cavities to near their normal size.

The mode of relative admeasurements here advocated, may also be employed advantageously for the determination of the different degrees of thickness of the cardiac walls and the ascertaining whether this is normal or not: for, if Nature accommodates the cardiac cavities to the extent of the aperture of the arterial orifices, it is but reasonable to suppose she would establish a relation between the size of this orifice and the thickness of the muscular substance which propels the wave of blood through it. As in the normal state, these orifices being narrow, and the cardiac cavities likewise small, the wave of blood, then of little volume, does not require such strong and thick muscular walls for its propulsion as in individuals in whom this capacity is larger. To ascertain the thickness of the walls of the ventricle precisely the extent of the capacity of its cavity must be taken into consideration: for by this alone can we clear up the questions of passive dilatation and concentric hypertrophy. This subject being, however, only secondary to the one whose illustration he had in view, M. Beau has as yet made none of the necessary measurements, and we need not therefore follow him in the interesting speculations he enters into respecting it.

In the categories already described, the *diameters of the orifices of both the aorta and the ventricle were found to be more considerable in proportion to the age of the subject;* confirming as far as they go the law laid down by M. Bizot, that the calibre of the heart and arteries, and the thickness of the cardiac and arterial parietes indefinitely increase with age, so as to be found at their height in old persons. M. Bizot employs this conclusion as a means of attack against *à priori* reasoning, by exposing the powerlessness of theory in reconciling this excess of material energy of the heart and arteries in the aged with the physical and moral debility which is their attribute: but the difficulty is not perhaps so inexplicable as M. Bizot deems it; for a dilated state of the heart and arteries may easily be conceived to be connected with general debility of the entire organism. Thus in old age we find the veins, especially of the extremities, the sphincters, and the aponeurotic rings lose their elasticity, or rather tone, and yield to forces tending to increase their diameter; and this same atony leads to an augmented capacity of the heart and arteries. The simultaneous increase of the thickness of the heart and arteries, takes place as admitted by M. Bizot in the intestines, stomach, or any organ submitted to dilatation; and did not the heart and vessels acquire from this cause some additional force, they would be powerless for the movement of the increased mass of blood their dilated condition admits the entrance of. This increase of substance is, however, in its nature, a morbid phenomenon, and not to be likened to that which occurs at an early period of life. In the latter, the normal increase of substance takes place alike in all the constituent parts of the heart, and especially at the semi-lunar valves. But, in the aged, these valves do not increase in extent simultaneously with the arterial orifices, and are not consequently sufficiently large to prevent some reflux of blood into the heart. There is, in relation to the cause, the greatest possible analogy between the amplification of the heart as a consequence of old age, and that which is developed in diseases characterized by the presence of arterial murmurs; since in both cases it depends upon atony arising from a relaxed condition of the muscles of animal life. There are these differences however—the amplification taking place in the aged very slowly, the hypertrophy resulting is far more considerable than in the pathological amplification; and, as in old age, the dilatation of the heart comes on so very gradually, the arteries have time to become proportionally enlarged: so that the normal relative capacity of the respective cavities is maintained. Consequently, though the wave of blood sent through the orifices is larger, the violence of the pulse of old persons fuller, and the dulness over the præcordial region greater, there is no production of the anormal murmurs, which manifest themselves in that pathological dilatation of the heart, which creates a disproportion between its cavity and that of the aorta. Nevertheless, in the aged, any increase of dilatation due to a morbid cause may induce such murmurs, by destroying the relation; but the defective proportion necessary for their production exists less often in the aged than in the adult or adolescent, notwithstanding their greater liability to adynamic diseases.—*Archives Generales*, T. xiv., pp. 133—161.

[We consider the above a very interesting communication, and forming a worthy complement to the elaborate series of papers referred to. The mode of comparative admeasurement suggested is the only one that can convey any precise idea of the capacity of the heart in relation to the functions it has to execute. —*Rev.*]

---

### Eschars over the Sacrum.

From the earliest period of his medical career M. Blandin has always entertained most serious fears for patients in whom sloughing over the sacrum occurs; and in his *Anatomie Chirurgicale*, published in 1826, drew the attention of the profession to the almost sudden manner in which they often prove fatal. He believes he has discovered the explanation of this in the following circumstances. The point which suffers most from pressure in dorsal decubitus corresponds to where the sacrum is joined to the coccyx—exactly there, where the vertebral canal is only formed by the posterior sacro-coccygean ligament. Sphacelus in this way may easily reach the termination of the arachnoid membrane, and air, pus, or sanies gain admission into its cavity, producing a violent inflammation, which at first attacks the nerves of the cauda equina. Necrosis, too, may open a way into the vertebral canal with the same results; and in both cases the phlegmasia which results induces the phenomena of paralysis of the rectum, the bladder, and the lower extremities. "When I made my earliest observations I was in the medical wards, and the accident is of no unfrequent occurrence in typhoid fevers. You have observed in the patient who has given rise to these remarks (a case of amputation of the thigh otherwise proceeding favourably) retention of urine and paraplegia."—*Gazette des Hôpitaux*, No. 71.

---

### Painful Crepitation of the Tendons. By M. Velpeau.

The man whom you have just seen is a dyer by trade, æt. 49, and his case deserves a moment's notice. A week since he endeavoured to raise a load, having his left hand applied to his hip. He felt a violent pain in this arm, and now we may perceive a slight swelling at the lower and external part of the forearm, unaccompanied by any change of colour or fluctuation. Of a regular and elongated shape, it is only painful during motion, while on applying the hand over it we may perceive a fine, characteristic crepitation; and it is an example of the *painful crepitation of the tendons* which was vaguely indicated by Boyer and Desault, described by me first in 1825, and has since formed the subject of the special writings of several authors. I first met with it in a case in the hospital of Tours, where it was suspected to be a fracture of the radius. The affection is especially observed among washerwomen, mowers, blacksmiths, locksmiths, and joiners, and when it is seated in the foot, among soldiers, huntsmen, &c. Excessive friction is the condition necessary for its production. In the forearm and wrist, where it is especially met with, its recognition is very easy, the crepitation it gives rise to being quite pathognomic, being neither like that felt in fractures, that of cartilage or emphysema : but which has been compared to the crepitation of starch or of hoar-frost—such as is produced by walking on the snow. Its seat is evidently the sheath of the tendons, and it is probably due to a slight inflammation, first causing too great a dryness of the mucous membrane, and afterwards giving rise to effusion. It is generally in no-wise serious, disappearing in a few days by rest alone : but it must not be absolutely neglected, for I have seen it in some cases give rise to a fungous transformation of the sheaths ; and indeed there is no reason why all the changes which occur in diseases of the joints should not take place here. If there is much pain we apply leeches and poultices, and the resolvent lotions and compression : but rest is indispensable.—*Gazette des Hôpitaux,* No. 82.

---

### M. Royer-Collard on the Statistics of Insanity.

In a Report to the Academy of Medicine upon an Essay by M. Baillarger, entitled " *Statistical Researches upon the Hereditariness of Insanity,*" M. Royer-Collard made some interesting remarks upon the undue importance given to this description of statement, a few of which, as having a far more general bearing than upon the case eliciting them, we here quote.

M. Baillarger's figures are as follow : they relate to 600 patients. Of 453 attacked by insanity in the direct line, the disease was transmitted 271 times by the mother, 182 by the father. Of the 271 families, in which it was transmitted by the mother, it occurred 70 times in more than one child, *i. e.* in more than a fourth : while of the 182, in which it was transmitted by the father, this was the case only in 30 instances, *i. e.* in one-sixth. In 346 cases in which the mother was mad, 197 girls and 149 boys were affected ; and in 215, in which the father was mad, 128 boys and 87 girls were so. M. Baillarger arrives at the following conclusions :—1. Insanity of the mother, in relation to hereditariness, is more serious than that of the father, not only because it is more frequent, but also because it is transmitted to a greater number of children. 2. The transmission from the mother is more to be feared for the girls than the boys, and, on the contrary, that proceeding from the father is more to be feared for the boys. 3. The transmission from the mother is generally to be more feared for the boys than that from the father : but it is to be much more feared for the girls."

" If the choice of the subject merits nothing but praise," observes Royer-Collard, " I must be allowed to express some objections to the method by which M. Baillarger has pursued it. These objections he has indeed foreseen, declaring that his observations are as yet but insufficient, and stand in need of completion. But I do not hesitate to say that it is Statistics which is guilty of this imperfection. In the eyes of many, Statistics, which it has been so improperly attempted to raise into a science, is of all our methods of scientific investigation the most positive and certain. So much have what are called facts been of late abused, that their very images have been elevated into a worship, or rather an idolatry. Figures for some, and even enlightened, men, are an irrefragible expression of the truth. But, let us not be deceived here : in the sciences, as elsewhere, figures, as well as words, have only a representative value. Statistics, which assembles them together and manœuvres them, is, of itself, blind and unreasoning ; so that, to reach that truth which it promises us, we must absolutely penetrate all this drapery, and proceed straight to the things represented through the signs representing them."

After alluding to the well established general facts of the hereditary character of the physiological and pathological condition of the economy, and of insanity among these latter, M. Royer-Collard observes that error and difficulty surround the subject when we attempt to submit this to arithmetical precision. In the first place, how are we to determine the hereditary relations of insanity? Admitting that we may be able to determine the insanity of individuals submitted to our examination; it is far from being always the case when we try to appreciate the mental condition of those we have never known. Where are the limits which separate sanity from insanity? A man has manifested peculiar tastes or strange ideas, without ever becoming mad. His son is manifestly insane, and will you call this hereditary or not?

"Another source of error deserves consideration. It is not only mental alienation, properly so called, that may induce insanity. All kinds of cerebral lesions, various nervous affections of uncertain nature and seat, certain congenital diseases of the organs of the senses, may exert the same influence. I may cite especially Epilepsy. What disease has more affinity with insanity, or is more frequently hereditary? Then again there is hysteria, hypochondriasis, and even that exaggeration of the general state of the economy termed 'the nervous temperament.' Apoplexy itself, and the habitual disposition to cerebral congestion, may easily, in transmission from parents to children, become in these a hereditary cause of insanity. The latter is only the result, the consequence of the original disposition which the son has inherited—only the chance of circumstances has terminated in him what nature had commenced. An accidental disease, a violent moral commotion, a change of climate, may determine in him the invasion of insanity, which the same would have doubtless also have caused in his father. Lastly, there is the singular fact of some diseases which do not seem at first to tend towards any particular condition of the brain, and yet do produce, either directly or by hereditary transmission, an entire alteration in the intellect. Pellagra, a disease as strange as it is terrible, almost always gives rise in succeeding generations to a description of insanity hence named pellagrinous. So, too, physicians of lunatic asylums know how frequently ulcerations are met with near the ileo-cæcal valve in the melancholic insane having a propensity to suicide. And who can say that this is not one of the tortuous paths by which insanity is transmitted from parent to child.

"I had occasion to consult upon this subject one of the men whose opinion carries most weight in question of mental disease, Dr. Calmeil, and this is his reply. 'During 15 years I took the greatest care in interrogating those who brought their relatives to Charenton. I insisted much upon questions relating to hereditariness, and went into the minutest details. I noted apart all that might some day throw light upon this important fact. But, when I had during several months, a year or more, continued to observe, to question, and to verify the exactness of the accounts which had been furnished to me, I discovered eight times out of ten that I had been deceived by the ignorance of some, and the voluntary falsification of others. Every year I was thus obliged to undo my work and begin again, until I have finished by abandoning it.' "—*Bulletin,* T. xiv., 760—776.

---

### M. Civiale on the Statistics of Lithotrity and Lithotomy.

M. Civiale recently read a paper upon the Value of Lithotrity before the Academy, in which he detailed the results of his large practice. These are thus briefly summed up. From 1824 to 1836 he had been consulted by 506 stone patients, of whom 307 were submitted to lithotrity, and 199 were considered as unfit cases. Among those lithotrized 9 were between 7 and 20 years of age; 55 between 20 and 40; 105 between 40 and 50; and 138 between 60 and 80. The number of cures amounted to 296; incomplete cures to 3: and deaths to 7.

From 1836 to 1845, of 332 stone patients, 241 were lithotrized, and 91 deemed unfit. This gives a total of 838 patients, of whom 548 were treated by lithotrity. To these are to be added 25 operations on account of relapse; 8 combinations of lithotomy and lithotrity; and 10 treated since the tables were drawn up; giving a general total of 591 cases in 22 years, 566 of whom were cured, 14 died, and 11 were more or less incompletely relieved.

He comments at length upon the disingenuousness of the adversaries of the new operation, who, in their criticisms upon the publication of the first portion of the above table, chose to comprehend in their enumeration of his failures the cases which he had declined to operate upon as unfitting. Out of the 838 stone patients, there were 290 in this predicament; and he observes that surgeons have not hitherto paid attention to this element in their calculations. In a statistical table which he long since published, containing 5,900 cases, in 859 lithotomy was not practised, and in 595 others all indications are absent; so that from the 5900 we have to abstract 1434. Most lithotomists have indeed omitted to keep any account of the numbers in which the operation was either declined or considered unjustifiable.—*Bulletin,* T. xiv., p. 821.

At a subsequent sitting, after referring to the various statistical tables of the results of Lithotomy: he thus states his appreciation of the two operations:—"1. That lithotrity well performed, and within the limits of its legitimate application, saves 96 or 98 patients per cent. 2. That about a fourth part of stone cases which are refractory to lithotrity may be treated by lithotomy. 3. That by lithotomy employed exclusively, and without distinction of age, from 20 to 30 patients per cent. are lost. 4. Applied to children alone, it saves nine-tenths. 5. Applied to adults and old persons, it saves from 50 to 75 per cent."—*Gazette Medicale,* No. 34.

---

### On the Induction of Cystitis and Albuminuria by Blisters.

M. Morel-Lavallée communicated to the Academy of Medicine the results of his researches upon the induction of Inflammation of the bladder by the use of Cantharides. These are, first as regards the *Etiology*. 1. The mode of preparing the blister is indifferent. 2. In general the action is more certain and more marked in proportion to the size of the blister; although the author has seen a blister not larger than half-a-crown placed upon the forehead determine the production of a false membrane of the bladder. 3. The distance of the topical application from the bladder is a matter of indifference. 4. It may happen that, upon the same subject, one blister produces no ill-effects upon the bladder, and yet, after a short interval, another of precisely the same size and make, re-acts powerfully on that organ. Generally, it is the last one which produces the inconvenience, though it may have been placed in quite another place, in which the epidermis offers the same defensive characters. Is this because the progress of the disease, which renders the topical application necessary, induces a debility favourable to absorption? 5. The preventive power of camphor is illusory.

*Symptoms.*—1. *Albuminous Secretion.*—The albumen may exist in three states: 1, in that of solution; 2, as a deposit at the bottom of the vessel; and 3, as false membrane forming in the bladder. The albumen existing in a state of *solution* is infinitely more abundant than in Bright's disease. In exceptive cases there are mere traces of albumen, but then the symptoms referable to the state of the bladder, as pain, &c. are few or none. In the third stage of the affection the *false membranes* vary in thickness from that of the merest pellicle to that of half a playing card. Sometimes they are rejected in such considerable rolls as to require traction to aid their expulsion from the urethra. Albumen is always found dissolved when it also exists as a deposit or false membrane; but the deposit is generally absent even when false membranes are present. 2. *Functional Symptoms.* Sometimes there is no pain, &c., and the albuminuria is the only symptom of the action of the cantharides on the bladder; but generally there is frequent micturition, strong smelling urine, periodical pains, &c. &c.—*Revue Medicale,* June, p, 288.

M. *Bouillaud,* likewise, recently called the attention of the Academy to the frequent production of albuminuria by blisters. He stated that, for a long time past, he had detected albumen in the urine of persons in whom no suspicion of serious disease of the kidney could be entertained. Recent observations have confirmed this remark, and have likewise shown him that the action of cantharides is capable of inducing its appearance. In the cases of patients admitted for other causes than Bright's disease, large blisters were laid upon regions of the skin wherein cupping-glasses had been applied. One of these was a man suffering from pleurisy with some effusion, whose urine tested had given no trace of albumen until after a blister had been applied, when it appeared in abundance—symptoms of strangury likewise existing. Blisters have since been applied upon eight or nine other patients, and in all of these next day albumen, which had not previously existed in it, was found in the urine (always most markedly so when the blisters had been applied to surfaces previously denuded of epidermis), tenesmus being likewise produced; and, in other patients who were blistered without any such effect on the bladder resulting, albumen was not found.

M. *Martin-Solon* observed that we should not confound a transitory albuminuria of this sort with albuminuria properly so called. In the first, the urine continues healthy, some albumen only being mixed with it; while, in the other, it undergoes a complete change, a description of combination resulting.

At a subsequent sitting, M. *Vernois* detailed the observations he had made confirmatory of those of Morel-Lavallée. Blisters were applied upon 26 men and 9 women, and albumen was found in the urine of 16 of the former and 3 of the latter. Strangury existed in 15 of the men and 3 of the women. In 7 of the men this existed without any coincident albumen. In two cases, in which the blisters were applied over scarifications, the pain and albuminous deposit were proportionally greater. In two ecchymoses, and in one phlyctenæ, were found on the bladder after death.—*Bulletin de l'Academie,* Tom. xiv. p. 745.

In reference to this subject M. *Miquel* has addressed a letter to the *Gazette des Hôpitaux* (No. 82) upon the best mode of preventing the irritation of the bladder here referred to. He says that M. *Bretonneau* and himself, after long experience, have found this to consist in not leaving the blister on the part too long. Four hours often suffice to produce vesication, and the intervention of a piece of tissue paper in no-wise impedes this.

He takes the opportunity of reporting upon the excellent effects M. Bretonneau has derived from the employment of *large flying blisters,* after local or general bleeding in rheumatism; these being applied to all the joints affected, so that three or four different ones have sometimes been blistered within the 24 hours. They are always taken off when the patient has complained of their irritation for 15 or 20 minutes, which he does in from 4 to 8 hours. Vesication may only follow the second dressing, and, if the blister has not been kept on too long and the epidermis has not been destroyed, the pain is very tolerable, and the healing prompt. M. Bretonneau has observed that when the skin has been repeatedly blistered, it at last ceases to vesicate, although there is nothing anormal discoverable about it. Small pimples on a red ground are produced by the blister, and if this be retained on, the bladder will become irritated though the skin do not vesicate. M. Miquel has found that a piece of the omentum of the calf or pig, which, if washed, will serve several times, forms the best *dressing* for blisters, especially if the epidermis has become torn. For the removal of the little pimples, furuncles, &c. which spring up around the margin of blistered surfaces, especially if these have been dressed with rancid substances, he finds the *sulphuret of potass* to form the best application, (1 part to 20 of water).

In relation to this subject we may here notice a new formula for a very active Blistering Plaister devised by M. *Mialhe.* After noticing the effects which cantharides sometimes produce on the urinary organs, he goes on to say—

"Upon what does this phenomenon depend? Doubtless, it must be referred to the absorption of the active principle—cantharidine: and to prevent its production we should put a stop to such absorption as soon as the desired local effect has been produced. Among the means suggested for preventing the specific action of cantharides upon the bladder, none is so good as that indicated by M. Bretonneau—the interposition of a piece of tissue paper soaked in olive oil. Cantharidine being soluble in fatty bodies, the oil serves as a vehicle for its introduction into the economy, but this introduction ceases to be active as soon as the serous effusion has taken place, since oily are not miscible with aqueous fluids. Another means consists in the association of *camphor* with the epispastic, but it attains its object less frequently than the other plan, notwithstanding that some practitioners consider it as infallible. Nevertheless, we believe the association of camphor with vesicating plaisters useful for two reasons. First, the abuse which has been made of camphor* of late has placed beyond all doubt the fact that this substance is endued with anaphrodisiac properties; and secondly, that, inasmuch as it possesses the power of softening the resinous substances contained in blistering plaisters, these become more fluid, adhere better to the skin, and consequently act more promptly. Now, we have assured ourselves by experiment that, all things being equal, the dynamic action of cantharides is so much the less to be feared as the local action is rapidly produced; or, in other words, that the absorption of cantharadine is so much the more marked as the serous effusion has taken long to produce. Hence we conclude: 1, That we should give the preference to the most active epispastic: 2, That vesicants should only be kept in contact with the skin for the time strictly necessary to effect a detachment of the epidermis; and 3, That camphor forms an advantageous addition to blistering-plaisters." The following is the formula for preparing an active one.

| | |
|---|---|
| Cantharides .. .. .. | 400 parts. |
| Lard .. .. .. .. | 25 |
| Veal Suet .. .. .. | 25 |
| White Pitch .. .. .. | 50 |
| Yellow Wax .. .. .. | 100 |
| Sulphuric Ether .. .. | 100 |
| Camphor .. .. .. | 40 |

"Powder the cantharides without previously drying them. Pass them through a silk sieve, and suspend the pulverization as soon as 100 parts of a fine powder have been obtained, which are to be placed in a wide-mouthed flask with the ether. Place the rest of the cantharides in a tinned pan with the lard and suet, and a sufficient quantity of water to cover them completely. Boil this gently for an hour, continually stirring it the while, and then let it cool in the pan. Next separate the fatty matter at the surface from the settling at the bottom, which last is to be thrown away. The fatty matter collected is to be melted without

* Camphor, under the auspices of that arch-quack M. Raspail, has obtained as much vogue among some classes of our neighbours as have Morrison's Pills, and other nostrums among ourselves.

water, and strained through a cloth into a tin water-bath, the pitch, wax and camphor added, and the whole heated until complete fusion takes place. Lastly, add the cantharides prepared with the ether, heat until the ether is entirely evaporated, that is to say for about an hour, pour the plaister into a marble mortar, and stir it until entirely cold.

"The effect of this vesicant is very prompt, taking place in from two and a half to three hours, according to the susceptibility of the cutaneous tissue, the more or less elevation of temperature of the part, and the closeness of the adhesion of the plaister. It offers some analogy with the *Emp. Cantharid.* of the London Pharmacopœia, which is well known to be very preferable to that of the Paris Codex; but it is more active, as we have assured ourselves by repeated trials: and this because it contains a larger proportion of the fly; and because all the blistering principle contained in it exists in a state of perfect solution, by reason of the manipulation it has been subjected to."—*L'Union Medicale,* No. 22.

[Judging from the frequency with which the subject of strangury following the application of blisters is noticed in the French writings, we suppose it is a matter of common occurrence. In our own practice we seldom or never meet with it, which perhaps may arise, in some degree, from our always directing the early removal of the blister. Still, among the common people, it is no uncommon occurrence to leave a blister on for from 12 to 24 hours, long after the epidermis has been raised or even ruptured by the fluid effused beneath it, and yet strangury is rarely met with.—*Rev.*]

---

### On Abscess of the Breast. By M. Velpeau.

"Subcutaneous inflammation of the Breast proceeds much as an ordinary phlegmon. When the abscess is formed between the mamma and the chest, the swelling is considerable, the breast raised up, but after an incision the cure usually takes place rapidly. But when the phlegmasia invades the substance of the breast itself, it is rare to find only a single abscess produced. We sometimes see 10, 20, 40 or 50 manifesting themselves in succession. An instant's reflection will show that this result is a natural consequence of the anatomical disposition of the inflamed tissue. The glandular parenchyma consists of different lobules, each of which constitutes a little organ having its own function, and which may become heated and irritated under the influence of lactation. Each lobule does not attain at the same time the same degree of irritation. One first inflames, then suppurates, and constitutes a first abscess: a neighbouring lobule then becomes affected and, in its turn, forms an abscess; and so it may go on with all of them until we have as many successive abscesses as there are lobules.

"This distinction of abscesses of the breast into at least three orders is of the highest importance; and if we do not adopt it, our ideas upon the subject will be but very vague, and devoid of all precision as respects prognosis and treatment. Parenchymatous abscesses may last four or six months, or a year even, according to the rapidity of their succession and their number. The subcutaneous abscess lasts only as long as an ordinary phlegmon; and the submammary abscess has not the long duration of the parenchymatous one.

"Each of these has again its special treatment. We may endeavour to procure the resolution of *subcutaneous abscess,* and that by ordinary means: and, if suppuration occurs, we open it promptly, in order to avoid the burrowing of the pus among the tissues. *Sub-mammary phlegmon* should be treated especially by general measures, and leeches around the nipple. Topical applications are of little use, as they are separated from the centre of inflammation by the whole substance of the mammary gland. When an abscess is formed here, its prompt evacuation is desirable: but the perception of fluctuation is difficult, for the pus is surrounded by a large mass of tissues, and the thoracic parietes have not fixity enough to serve as a point of support. Nevertheless, you may recognize the existence of pus by the following characters. 1. An acute phlegmon rarely exists more than seven or eight days without suppuration taking place. 2. The breast is raised up like a sponge, and if we press upon it, it seems as if it were lying on a bladder full of fluid. 3. We find the breast surrounded by a kind of inflammatory œdema. Having recognized the pus, we should let it out promptly, or we expose ourselves to seeing it traverse the gland and form one of those abscesses I call *shirt-buttons.* These abscesses, moreover, have a mischievous influence upon the chest, and may lead to a purulent pleurisy. They may, too, penetrate into the cellular tissue for a distance, and give rise to a diffused phlegmon. The incision should be made into the most dependent part, the place of election being below and at the outer side of the nipple; but, in some cases, a projecting point of the abscess indicates the place at which the opening should be made. It is always advantageous to make the incision towards the circumference of the breast, because the gland itself is not touched, and its weight tends to expel the pus. The bistoury should be directed almost parallel with the thoracic parietes, so as to slide it in between these and the mamma. The danger of such incisions is not great, there being no large arteries to fear. *Parenchymatous phlegmon* requires an energetic and varied treatment. Bleeding, purging, and the so-called anti-lactal medicines. When pus forms, which is almost always the case, topical applications and incisions seldom prevent the successive implication of the lobules. Nevertheless, there is some advantage derived from the prompt opening the abscess, *if the patient agrees to it;* for you should recollect that, in practice, if you open one abscess and others form, she never fails attributing these to your proceedings. These details will, I think, suffice to show you how important it is to distinguish the different abscesses of the breast, and to explain to you the confusion which prevails in the minds of some surgeons as regards their treatment."—*Gazette des Hôpitaux,* No. 89.

[The above remarks, like all that falls from this excellent surgeon, are well worthy the attention of the practitioner. We have seen much needless suffering caused by the formidable operation of incising through the whole substance of the breast, to get at an abscess situated at its posterior part, and which might have been much more easily reached by the circumferential incision here recommended.—*Rev.*]

---

### On the Nature of the Liquid secreted by the Mucous Membrane of the Intestines in Cholera. By M. Andral.

M. Andral recently read a note at the Academy of Medicine, giving an account of the researches he has been engaged in for the purpose of determining the nature of the peculiar white matter, resembling a decoction of half-cooked rice, which is found in the digestive organs of patients attacked with cholera, and which especially belongs to and characterises that affection. From the facts detailed he drew the following conclusions:

"1. The white matter which fills the intestines of cholera patients is not, as it has often been stated to be, a portion of the blood itself; for neither albumen or fibrine are found in it. 2. It is nothing else than mucus rapidly secreted in large quantities, and for this reason modified in its qualities. 3. The essential microscopic character of this matter is its containing a very considerable number of cells, with nuclei perfectly resembling, as far as regards their appearance, the cells found in pus, although this matter in no other respect bears any resemblance to pus. 4. The examination of the blood of cholera patients shows that the albumen of the serum is maintained in its normal proportions. 5. The theory which refers the symptoms of the stage of cyanosis in cholera to the change which the blood has undergone by reason of a great and sudden loss of serum, cannot be admitted."—*Gazette Medicale,* No. 33.

---

### Induction of Premature Labour for the Prevention of the Death of the Infant in Miscarriage at the Eighth Month.

Dr. Secondi proposed the following question for the consideration of the Medical and Surgical Section of the Eighth Italian Scientific Congress, held at Genoa, Sept. 1846. "*Whether, in the case of several successive deliveries of a dead fœtus in the course of the eighth month, it is not admissible in a consecutive pregnancy, to induce premature delivery at the seventh month.*" In support of the proposition he narrated two cases observed by him; one of a lady, who was delivered in eleven successive pregnancies of dead children at the eighth month, although the fœtus had continued alive after the seventh, and every means that could be suggested by art, and these of different kinds in the respective pregnancies, had been put into force in order to avert the catastrophe; in the other case, the lady miscarried in this way five times. In both these cases the position of the patients was such as to admit of their adopting every hygienic precaution recommended, their health seemed quite good, and they were taken in labour without any indications of pre-existing or present affections, the fœtuses and their dependencies likewise not manifesting any signs of disease. Reasoning upon these facts, it seemed to him that these women were governed by different vital laws, either primarily so, or secondarily dependent upon the presence of the fœtus, by reason of which the nutritive relations between it and its parent ceased, and it was separated just like a fruit from the tree. As the provocation of premature labour does not compromise the life of the woman, and very little endangers that of the child, he answered the question in the affirmative.

Dr. *Arata* supported this conclusion, and related two cases in point. In the one, the accident in question had occurred eleven, and, in the other, five times, in spite of the employment of every kind of therapeutical agency; and notwithstanding that the subject of the last case was of a favourable temperament and in good health, and the other, in consequence of a suspected syphilis, had been duly treated. He added that the fœtuses possessed a due development, with no indication of disease in them or their dependencies; and that if we induced premature labour on account of a narrowed pelvis, we should do so also in the case in question.

Professor *Centofanti,* although inclining to Dr. Secondi's opinion, would first wish to learn the cause which prevented the fœtus continuing to live after the eighth month, and whether it died really in the eighth month or before. The causes which lead a woman to abort in the eighth month must be either inherent in her own organization, or in that of the fœtus or its dependencies. He believed that the causes inherent in the woman, if not organic, may be removed by a properly adapted method of cure; but that such is not the case with those dependent upon vices of the fœtus or its dependencies. Where, too, they depend upon fœtal disease, before we can determine whether we can or not prevent the bringing forth a dead fœtus, it would be requisite to examine whether these all were derived from the fœtus, or from the special condition of the woman reflected back upon the fœtus. Lastly, it is to be recollected that a fœtus may die at the fifth or sixth month, and continue to remain in the uterus until the eighth, providing the membranes have not been ruptured: so that the propriety of the operation is confined to those cases in which we are certain that its death takes place in the eighth month.

Professor *Vannoni.* We should look at this question in its true aspect; the death of the child. What ought to prevent us endeavouring to save an infant which would otherwise die at the eighth month? And why is the Cæsarian section or symphysisotomy instituted but that the fœtus has a right to social life? He considered the question of the cause of the death of the child at the eighth month as one of the highest moment, and besides the recognized organic causes, and the maternal or fœtal diseases, &c., he would take into account other —*physical*—causes. It does not suffice for the explanation of these accidents to invoke a particular condition of the woman. There are other causes on the side of the fœtus. We should bear in mind that the fœtus, having reached a certain point, can live no longer, although the mother is healthy, and it itself, after expulsion, exhibits no mark of disease: and we must admit here an intrinsic cause which operates within the uterine cavity, by reason of which the fœtus has reached such condition of development at the eighth month, as to be already old and unable longer to retain life. When, therefore, the ordinary means are at fault, and a careful diagnosis has been made of the causes which could perchance give rise to this accident, premature labour should be induced. But are we certain, by this means, of preserving the child's life? To decide this we have need of experiments, and in inducing premature labour even in the case of our not succeeding in saving the fœtus, we have removed it from the cause of death to which it was exposed.

The Section eventually came to the following decision:—*That when, on four or five successive occasions, a woman has been delivered of a dead fœtus at the eighth month, it is justifiable in future pregnancies to induce premature labour.*—*Annali Universali,* Volume 121, pp. 172-5.

[This application of the induction of premature labour is new to us, and one of very doubtful expediency. The question of the cause of the death of the *fœtus in utero* is involved in too much obscurity to admit of positive opinions upon many points being pronounced; but it seems to us, when we bear in mind the great difficulty in preserving and rearing premature children, that one whose vital powers were so limited as not to admit of the retention of life *in utero,* would have its chances of so doing little if at all increased by inducing its more hasty expulsion. Admirable as is this resource in appropriate cases, and foremost as have the practitioners of this country shewn themselves in employing it, the induction of premature labour is not an operation to be extended to additional emergencies without the gravest consideration and the strongest probabilities of success. Independently of the danger of too hasty a generalization of it in a moral point of view, it is by no means so unattended with risk to the mother as our Italian brethren (prevented by priestly interference from employing it in appropriate cases) would seem to infer.

We may take this opportunity of expressing the great pleasure we have derived from the perusal of the proceedings of the Medical Section of the Congress. Several excellent and learned papers were read, the spirited discussions upon which well proved that the Italian physicians and surgeons were as earnest participators in the progress of medical science as their brethren of any other portion of Europe. This Section seems to have taken a prominence in the proceedings of the Congress not observed in the scientific periodical assemblages of France and England; which is probably due to the far more rapid diffusion and interchange of ideas which takes place by means of the organs of the medical press in these two latter countries than in Italy—where discussion of any kind is but a thing of yesterday.—*Rev.*]

### Purulent Ophthalmia of New-born Infants.

M. *Chaissaignac,* surgeon to the Foundling Hospital at Paris, has recently addressed a letter to the *Académie des Sciences,* giving some account of the views he entertains concerning this affection, and of the success which has attended his treatment of it by irrigation. Since then, M. *Laborie* has attentively examined the subject in M. Chaissaignac's ward, and furnished the following account for the pages of *L'Union Medicale.*

When M. Chaissaignac received his appointment to the Foundling Hospital, he was struck with the great number of children who were suffering, in different degrees, from the consecutive effects of purulent ophthalmia. Attentively examining the disease for the purpose of endeavovouring to prevent the continuance of such calamitous results, he discovered that this inflammation presents a characteristic which has hitherto escaped observers. With very few exceptions, a *pseudo-membrane* was found to be present on the conjunctiva, the removal of which was found much to facilitate the successful issue of the disease. This assimilates the disease to various other affections frequently met with in infancy, such as muguet, diphtheritis, croup, &c. This result, to which M. Chaissaignac's direct observations led him, appeared to us to be beyond all doubt; for we had already remarked during our residence at the *Clinique,* a remarkable and almost constant coincidence between the appearance of muguet and purulent ophthalmia. So constantly did these affections show themselves one after another that M. Paul Dubois always predicted to us, when he had observed some cases of muguet in the wards, that we should soon see examples of purulent ophthalmia.

In his letter to the Academy, M. Chassaignac reports the great success which has attended his mode of *treatment by irrigation,* and it will be as well to state what induced him to adopt this, and the mode in which he employs it. Being in charge of the *Hôpital de l'Ourcine,* he had a great number of women under his care who were suffering from *vaginitis* attended with the most obstinate discharges which every form of the ordinary treatment, perseveringly tried, failed to subdue. Believing this to arise from portions of the mucous membrane which the applications had not reached, constantly secreting a contagious liquid, it struck him that keeping these parts constantly washed might lead to more fortunate results. Since this has been put into force far greater success has attended the treatment by medicated injections, &c.

In purulent ophthalmia, also, the remaining portion of the mucous membrane is much rather affected by contagion than by continuity of surface, and *constant irrigation* seemed the most effectual means of preventing this through its prompt removal of the contagious matter. M. C. commenced his experiments very carefully, until he had ascertained that the means was positively harmless when not efficacious. The following is the manner in which the irrigation is employed. A reservoir is fixed against the wall at about three or four yards from the ground, having two cocks at its lower part, to which are adapted caoutchouc tubes, with apertures of about 2 *millimetres* diameter. By means of a moveable diaphragm the force of the stream may be regulated. The child is laid upon a table covered with waterproof cloth, with its head placed against the wall under the reservoir, and slightly inclined backwards, so that the rest of the body is not wetted. The two tubes are brought to a level with the head, and the operator directs the jets first upon the eyelids, then against their edges, and after some minutes, over the entire conjunctiva. The operation is continued for 10 or 15 minutes or longer, and is repeated several times a day. Generally, the child manifests no sign of suffering as long as the eyelid is only watered, and sometimes not even when the interior of the eye is; but for the most part it then complains, and, if the operation is too prolonged, becomes very impatient under it, and cries, so that a suspension becomes necessary. During the operation the eye becomes a little red, and all the secretions and pus are removed from the edges of the eyelids. When the fluid pus has been thus removed also from the mucous membrane, the conjunctiva is found of a greyish aspect, which seems to result from a purulent deposit. But in spite of washing, this does not disappear, and it is in fact the *false membrane* indicated by M. Chaissaignac, which, under the influence of the water becomes more opaque. It may be raised and removed without much difficulty by means of a pair of fine forceps. Sometimes no erosion of the surface is left by its detachment, but at others there is all the appearance of a true wound left. Sometimes the membrane is very extensive, covering all the ocular conjunctiva as well as the cornea. At others it is only partial, being seen at several unconnected points. When the irrigations have been employed and this membrane removed, it is not reproduced upon the same part. Astringent collyria are used during the day, although, since he has employed irrigation, M. C. seldom has recourse to these.

M. Chaissaignac has employed the irrigation in other affections of the eye with advantage, such as obstinate *blepharitis.* Of 76 patients admitted during the last ten weeks, 40 were suffering from well-marked purulent ophthalmia, 20 from various forms of blepharitis, 7 from papular conjunctivitis, and 4 from old opacity. The duration of treatment has, for the purulent ophthalmia, occupied a mean period of 10 days, for the conjunctivitis 4 or 5 days, and for the blepharitis 3 weeks. In no one of the cases of purulent ophthalmia did the cornea become affected.

M. C. does not attribute to the irrigation any great special medicatory power, it being, in fact, chiefly a hygienic measure for the removal of the abundant discharge: and collyria are employed while resorting to it. But under its influence the false membrane becomes more easily visible and consistent, and consequently more easily detached; the removal of this, which acts upon the eye with all the injuriousness of a foreign body, expediting the cure and rendering it more certain. The application of cold also, of such utility in other inflammations, doubtless has a beneficial effect in this.

In appreciating the success of this treatment we must not, however, overlook that also derivable from other procedures; thus, we have not met with a single case at the Clinical Hospital, during two years, which did not yield to the following means, viz. a collyrium of nitrate of silver (32 parts Aq. dest., $\frac{1}{10}$ part Nit. Arg.); a slightly emollient collyrium frequently during the day; great cleanliness of the parts to be secured by preventing the morbid secretions remaining in contact with the eye. In very rare cases a slight application by means of a pencil of nitrate of silver is made to the inner surface of the eyelids. M. Paul Dubois says he has never known an eye to be lost under this treatment.

We have already stated that M. Chaissaignac has treated several cases of blepharitis by irrigation with great success. Several of those we examined were examples of ciliary *blepharitis,* and the obstinacy of this disease is well known. A circumstance which contributes to the tediousness of cure is the manner in which the secretion from the Meibomian glands glues the eyelashes together, forming them into hard brushes which simple lotions can scarcely render supple. By means of irrigation, which produces no painful or disagreeable sensation, they are freed in some minutes from all concretions. They become supple and no longer stick together.

M. C. likewise believes that *staphyloma* may be advantageously treated by irrigation. He does not pretend to say it may be so cured, but, under the use of this means, he has observed a positive arrest in its progress, and sometimes even a slight diminution of the projection. The douches in this case seem to exert a resolvent and tonic effect.—*L'Union Medicale,* Nos. 108-109.

[When M. Chaissaignac's letter was read at the Academie, M. *Flourens* took the opportunity of stating that he suspected the so-called pseudo-membranes of the mucous membranes were nothing but portions of detached epithelium. M. *Velpeau*, while believing this may sometimes be the case, considered that it was not so in the present disease. Diphtheric inflammation is not confined to the mucous membranes, for it may affect any region of the skin; and, on its removal, this structure be found sound beneath.

Although we certainly have never expressly examined the eye for the detection of the false membrane in question, we feel somewhat sceptical as to its usually forming a characteristic of this disease. It may, perhaps, do so sometimes in the impure atmosphere of the Paris Foundling Hospital, but it could scarcely have escaped detection in private practice were it a common occurrence. We have frequently seen the discharge assume an almost membranous tenacity. In respect to treament, it seems to us that free syringing the eye with cold water would accomplish all the objects derivable from irrigation; but we would not like to employ this as any other than a subsidiary means. The hourly use of injections of sulphate of zinc or nitrate of silver generally rapidly subdue the disease, although the assistance of a leech may occasionally, but very rarely, be required also, if the inflammation, as denoted by the tumefaction of the lid, is excessive.—*Rev.*]

---

### On the Treatment of Typhoid, or Entero-Mesenteric, Fever by the Black Sulphuret of Mercury. By M. Serres.

M. Serres has recently read some papers upon this subject before the *Academie des Sciences*, and as they have excited much attention in Paris, a notice of some of the principal points dwelt upon by him may prove acceptable to our readers.

He believes that the symptoms, progress, and anatomical lesions of this disease all show that it belongs to the exanthematous fevers, and this fact constitutes the basis of the proposed treatment. The histories of measles, scarlatina, erysipelas, but especially of variola and vaccinia, prove that the amount of fever is proportionate to the amount of eruption; if this is discrete the fever is slight; if it is confluent the fever is intense; "it becomes confluent also by the change which takes place in the composition of the blood, and the phenomena of re-action which are developed throughout the system." As long ago as 1812, the author, together with M. Petit, endeavoured to demonstrate a like dependence of typhoid fever upon the amount of entero-mesenteric changes which were developed; and 35 years additional opportunities of investigating the subject at La Pitié and the School of Anatomy, where bodies are brought from all the hospitals of Paris, have conferred upon the proposition all the certitude attainable in medicine.

"If, as now stated, every eruptive fever is compounded of two distinct elements: of the eruption, which is the dominating element, and of the fever which is the dominated element, the therapeutical course is traced out in this disease by this subordination of the phenomena. Reasoning indicates this, and medical experience has demonstrated it. In the remarkably faithful picture drawn by Sydenham of the progress and generation of symptoms in the small-pox (the passages are quoted, but they are or ought to be familiar to our readers), we recognize that of the typhoid fever which was furnished by M. Petit and ourselves. If, in fact, in the comparison of the two diseases, we form an abstraction of the eruption or fundamental portion of each, we find a perfect resemblance in the phenomena of the consecutive fever constituting them—the same infection of the blood—the same permanence in the source of the infection—the same saturation of the system with a deleterious principle. The bases of their therapeutics should partake of and reflect this uniformity. But, for the bases of therapeutics to assume such conformity they must be able to extend to the foundation of these two diseases. And here is the difficulty. With respect to small-pox the etiology has never been contested. All agree that, beyond the affection of the skin, there is a general affection, having its vehicle in the mass of the blood. It is not the same with the etiology which is here given of typhoid or entero-mesenteric fever. Eminent observers, and whose consummate experience might well serve as a guide in medicine, have entertained an opposite opinion. They have seen in the disease only an enteritis, or an inflammation of the intestine, different degrees of which might explain the general and local symptoms by which it reveals itself."

"The treatment I propose consists in the administration of the *black sulphuret of mercury* in the form of pills, and the inunction of the abdominal parietes by means of the mercurial ointment every morning. Four grains of the black sulphuret are formed into a pill with tragacanth and syrup; and from four to six of such are given every second day. The treatment may be continued for six or eight days, provided no stomatitis occurs. If the mucous membrane of the mouth becomes inflamed the frictions are to be suspended and the sulphuret diminished or discontinued, applying alum gargles or slices of lemon to the gums.

"Although every one recognises that the gravity of the disease is dependent upon the amount of the intestinal eruption, no one has hitherto tried to treat this topically. Purgatives in general fulfil the first indication of treating the general poisoning of the system; but it is the mercurial purgative which alone exerts a special topical action on the intestinal patches. We cannot give proofs of this direct action; but from the effect which mercurials exert on analogical diseases, we are enabled to make an *a posteriori* induction upon the subject. We know that the application of mercury procures the abortion of variolous pustules. Mercurial frictions dissipate an erysipelas springing from internal causes—as they do the rose-coloured lenticular patches which appear on the abdomen in typhoid. The diarrhœa and distension of the abdomen in typhoid are certainly due to the irritation which the intestinal eruption determines upon the mucous membrane of the intestines; and both these symptoms are relieved under the use of the black sulphuret (although ordinary purgatives fail to relieve them), proving that this exerts a topical action upon the intestinal eruption, preventing or arresting its development. But it likewise exerts a more generally beneficial effect upon the organism, seeming as if it reached the cause of the disease itself;—the fever becomes less, the pulse diminishes in number, and the delirium abates—and this in so decided a manner as to be obviously the result of the medicine. By this method we do not abridge the duration of the fever. It continues, as under other treatment, for 3 or 4 weeks: but generally, when seen early, it is conducted through its course without any accident arising."

"Although the lenticular and rose-coloured spots on the abdomen, which constitute so characteristic a symptom of typhoid fever, differ essentially from the variolous pustule, yet the septic nature of the two diseases, the concomitant changes in the state of the blood, led me to the study of the action of mercury on these petechiæ. In the year 1845, this petechial eruption was remarkably abundant in most of these patients; but, under the application of the mercurial ointment, they disappeared very rapidly—the accompanying meteorism simultaneously diminishing. This double result led me to conclude not only that the mercury operated beneficially upon the petechiæ, but also upon the intestinal eruption, which constituted the foundation of the fever. If this last conclusion were correct, it was reasonable to suppose that, could the mercury be brought into direct action on the intestine, its effect would be still more prompt and efficacious; and, after an attentive examination of several pharmaceutical preparations of this metal, the black sulphuret seemed the best adapted to fulfil the desired indication."

Some particulars of a few of the cases which have fallen under M. Serres' notice are furnished, and he draws the following conclusions:—1. The fever and cephalalgia have been evidently influenced by the second or third day of the medicine. 2. The pulse has fallen below the mean, and even become remarkably slow. 3. No adynamic or ataxic accidents occurred; and, when adynamia appeared at the commencement of the disease, it was soon removed. 4. The quantity of ethiops employed to procure these results has not exceeded 50 grains, and several times but 30 have been administered. 5. Only a slight stomatitis, of which the patients hardly complained, was produced. 6. Convalescence was fairly established from the 8th to the 15th day, return to health always having been accomplished without relapse. 7. The patients left the hospital entirely cured in between 30 and 50 days; although they were encouraged to stay in as long as possible for the purpose of observing any relapse if such occurred.—
—*Gazette Medicale,* Nos. 33 and 34.

[That the consentaneous employment of the black sulphuret of mercury and mercurial inunctions have proved highly useful in typhoid fever, the statements of so intelligent an observer as M. Serres sufficiently prove; but that such effect has resulted from the ectrotic effect this substance has exerted upon the diseased follicles of the intestinal canal is the purest conjecture. Even in the analogical case of small-pox, so much dwelt upon in the memoir, mercury exerts no specific action in producing the abortion of the pustules, the same end being attainable less conveniently by the use of caustic and more conveniently by the application of blisters. Still, with any of these aids, the progress and result of the disease is capable of very slight modification, though, for the prevention of disfigurement of the face, they become valuable subsidiary means. We are not told whether the sulphuret acts as an active purgative. If so, its utility may to some extent be explained, as many of the French practitioners have derived great benefit from this class of medicines, mercurial and otherwise, now the belief in the disease being essentially an enteritis has nearly passed away.—*Rev.*]

---

### The Medical Profession in Paris.

According to M. Domangés Medical Almanack, the number of practitioners in Paris 1st Jan. 1847, amounted to

| | | | |
|---|---|---|---|
| | Doctors of Medicine .. .. | 1442 | |
| | Officiers de Santé .. .. .. | 175 | |
| | | | 1617 |
| To these may be added, | Pharmaciens .. .. .. | 345 | |
| | Midwives .. .. .. .. | 480 | |
| | Total .. .. .. .. | | 2442 |

In estimating the amount of practice which falls to the lot of these 1617 qualified medical men (at Paris as elsewhere, charlatans and unlicensed persons abound likewise), we find the official statements in 1846 give us a population (including, however, hospitals, colleges, and garrison) of 1,053,897 souls. In 1845, of 32,905 births, 5,678 occurred in the hospitals, that is more than a sixth part did not fall into the hands of the practitioners and midwives, these last taking the lion's share of the remainder. In the same year, 25,156 deaths took place, of which 15,888 took place at their own houses, the rest dying in hospitals, &c. In 1845 there were 1598 practitioners, and dividing the deaths among these, we find about 10 must fall to the lot of each. In the hospitals, the proportion of deaths to patients is about one-eighth; but, if in the easier classes of society, we call it a fifteenth, each practitioner would then, upon an average, have an annual practice among 150 patients, or 12 per month. We may leave to others to calculate the mean number of visits these may require, and the product of such, at charges of 5, 3, 2 francs, or even less—to say nothing of bad debts. This, if patients were equally divided; but there are some few of great repute who receive their £4000., others whom the hardness of the times leaves only £2000; and so on, from step to step, to those who, yet better provided for than the mass of their brethren, with great difficulty attain £200. at the end of the year. Lower, and much lower still, there are practitioners who make as little as £60. £50. or £40. per annum; and how many are there in the first year of their practice do not take £20.

And yet the evil is on the increase: the number of practitioners is constantly augmenting. In 1845 there were but 1430 doctors: 50 died and 62 left; but this vacuum of 112 was not only filled up, but replaced by 124. In 1845 there were 168 *officiers de santé,* 175 in 1847: so that the total addition of practitioners in only two years amounted to 19. In 1845 there were 450 midwives, in 1847 there are 480; while the accouchements at home in 1845 only amounted to 27,227. Take from these the gratuitous confinements provided by the charitable societies, and those conducted by the practitioner, and there certainly cannot remain a mean of 50 patients per annum for each midwife. The majority then cannot live by their profession. What then do they live by? That would be worth while inquiring into!—*L'Union Medicale,* No. 102.

[The writer goes on to recommend the limitation of the number of the practitioners to the wants of the population; forgetting apparently that every occupation and profession is in like manner overburdened and could put forth equal claims to repression, if these were not perfectly inadmissible in the present state of society, which is to be governed by moral and prudential restriction, and not by the exclusivism of a worn-out period. The medical profession has, however, the remedy in its own hands in a great measure, by means of which it may right itself, and yet benefit the public, namely, the increasing the period of study and the amount of requisite qualifications. Certainly the pecuniary position of the medical body in Paris, if the above representation be correct, is far worse than that of our own capital: since, for our own two million souls, we have but 2,413 qualified practitioners, while for their 1 million, they have 1617. Then again, the midwives with us are a most insignificant class, employed only by the poorest of the population as a matter of necessity; while too many of our own practitioners add to their strictly professional receipts the profits of tradesmen by vending drugs, perfumery, and even quack medicines! For our parts we prefer the honorable poverty of our neighbours to this lucrative but degrading practice among ourselves.—*Rev.*]

### Observations on the Materia Medica.

1. *On Alkaline Medicines. By M. Mialhe.*—M. Trousseau has recently recalled attention to the fact that, when these are taken in too great a quantity, they increase the fluidity of the blood and deprive it of colour, and lead to cachexia, pallor, and general infiltration, passive hæmorrhage, and an irreparable wasting—giving rise to far greater and more irremediable evils than the disease they are employed to combat, and causing just as much mischief as the abuse of iodine, mercury, or the preparations of iron. I propose here to indicate some of the circumstances under which alkalis may give rise to these ill consequences, and those of a contrary character, under which perseveringly used, they may re-establish the deranged equilibrium of the economy.

First, we may observe that the administration of alkalis in excess does not give rise to so much mischief as does a similar abuse of acids. In the state of health the three principal fluids of the body, the chyle, the lymph, and the blood, are alkaline, and the amount of alkaline base they contain is incomparably greater than the amount of acids contained in other fluids. It is then in an alkaline medium that the animal organic mutations are operated, while in plants it is always in a neuter or acid medium that the phenomena of nutrition take place. The alkalis fulfil a far more important function in the economy than that of mere exciters and fluidifiers, presiding as they do over the decomposition and assimilation of the hydro-carbonaceous substances of the amylaceous or cellular families.

But however the blood and other liquids may be physiologically a little more or a little less alkaline, it does not follow that they may be inconsiderately administered. They stand at the head of the agents which exert on the serum the most marked fluidifying action. All the alkalis affect our economy in an identical manner. They produce on the organ of taste an impression *sui generis,* designated as *alkaline* or *urinary.* M. Chevreul showed this to be always due to the same substance, ammonia, which is set free by the decomposing effect exerted by the alkaline base on the hydrochlorate of ammonia contained in the buccal fluids. Experience has shewn that, the same chemical fact prevails in respect to the bicarbonates and carbonates, and all the other fluids of the living body. Hence, wherever we introduce any alkaline substance into the economy, a certain quantity of ammonia is set free. This explains why the ingestion of a certain quantity of bi-carb. soda dissipates the symptoms of drunkenness—the ammonia disengaged restores to the albuminous elements of the blood that fluidity which the coagulating action of alcohol had partially deprived them of.

Under what circumstances is the employment of alkalis efficacious or dangerous ? Clinical observation shows us that the daily taking of a drachm or a drachm and a half of bicarbonate of soda, or its equivalent of any other alkali, so far from being generally injurious is frequently advantageous. Many persons can take far larger doses with impunity, while much smaller ones have, in some cases, induced serious accidents. All substances which produce an acid predominance in the blood allow of a large quantity of alkalis being taken. Thus, the inactive inhabitants of towns, in whom there is hardly any acid secretion from the skin, especially in Winter, will bear large doses of alkalis. It is the same with those who live upon an almost exclusively meat diet, inasmuch as the albuminised elements containing sulphur and phosphorus, these two elements produce, by their interstitial combustion, phosphoric and sulphuric acids in marked proportions. This explains why the urine of the carnivora is normally acid, that of the herbivora always alkaline. On the other hand, whatever favours the predominance of alkalis in the vital humours forbids their employment. Thus the laborious inhabitants of the country, in consequence of their abundant acid sweats, can ill bear the ingestion of alkalis. So with persons who adopt an exclusively vegetable regimen, the blood is normally rich in carbonate of potass, by means of the transformation which the salts of potass in combination with the organic acids have undergone. Lastly, there are certain pathological conditions which lead us to vary the amount of alkalis given. Who does not know that in gout, gravel, and especially diabetes, immense quantities of these may be given ? and who is not aware that certain Summer and Autumn putrid affections will not tolerate them ?

Clinical observation has long shown that the various alkalis may replace each other in practice. This is the case with carbonate of lime and of magnesia: as also with the compounds of soda and potass. It has been erroneously stated that, soda is more favourable to the animal constitution than potass. Analysis of the animal liquids shows that the potass compounds are equally prevalent with the soda, and as regards the herbivora, far more so. But, although we can in general by the aid of any alkaline preparation induce an identical medical result, we believe it is better to give the preference (as mere antacids) to those alkaline compounds which present the advantage of having always an uniform chemical composition, and of producing little or no therapeutical effect; and in these respects the hydrated calcined magnesia and the bicarbonate of soda seem to hold the first rank.—*L'Union Medicale,* Nos. 1 and 4.

2. *Alum Gargles.*—We have shown elsewhere that the true *astringents* belong to the class of *coagulants,* that is to say, the class of chemical agents capable of entering into chemical combinations with the albuminous elements of the blood, and forming with them an insoluble compound. In applying this principle to *alum,* we showed how this substance, penetrating into our tissues, is first decomposed by the alkalis of the blood, so as to form an insoluble sub-salt, which is deposited in the organic tissues, filling their network, and, so to speak, tanning them. We pointed out how a new portion of alum, being no longer modified by the alkalis already saturated, then acts, by fluidifying the albumen, in stimulating exhalation; and how, lastly, this *alum-albuminous fluid,* taken into the circulation, again becomes solid when it finds itself in presence of all the alkalis contained in the mass of the blood—and in this way we explained the agency of large doses of alum in arresting hæmorrhage. Thus, in a small quantity it is a very precious local astringent; in a larger quantity it becomes an energetic local fluidifyer; and, after absorption, a general hæmostatic of undoubted efficacy

We omitted to state why we thought this substance should not be combined with *mel rosæ,* which it so commonly is. It always contains a marked quantity of the proto-sulphate of iron by reaction on which the tannin of the mel rosæ produces a greenish precipitate. This, besides being disagreeable in appearance, leads the patient to the belief that the properties of the medicine have become deteriorated. Preferable combinations are the following:—

1st. *Astringent Gargarism.*

| | | |
|---|---|---|
| Alum .. .. .. .. .. .. | | ½ a part. |
| Distilled water .. .. .. | | 150 parts. |
| Syrup of mulberries<br>——— Poppies, | of each | 25 parts. |

For aphthous affections, mercurial stomatitis, and generally in all the diseases of the throat in which astringents are indicated.

2. *Detersive Gargarism.*

| | | |
|---|---|---|
| Alum .. .. .. .. .. .. | | 20 parts. |
| Distilled water .. .. .. | | 100 parts. |
| Syrup of mulberries<br>——— Poppies | of each | 30 parts. |

In hoarseness, in aphonia, and in those affections of the pharynx characterized by great dryness, and in which it is desired to excite the excretion of the mucosities. It is in this same dose—that is its fluidifying dose—that alum should be given for the prevention and cure of pharyngeal diphtheritis.

Most practitioners, I may remark, administer alum in too small a proportion for the removal of acute hoarseness, and especially in the aphonia of singers. Theory and practice alike show us that these cases require strong gargles.—*Mialhe, l'Union Medicale,* No. 34.

3. *Astringent Collyria.*—Many practitioners are in the habit of prescribing *mucilages* of gum, psyllium, and especially of quince, with the different medicinal agents which constitute the base of their astringent collyria. It is a bad practice, since all true astringents necessarily belong to the class of bodies which coagulate the serum of the blood, and all substances which coagulate albumen also coagulate gum and the liquids which contain it; whence it results that the addition of mucilage to a salt of alum, zinc, copper, lead, silver, &c., necessarily gives rise to more or less of an entirely insoluble precipitate, which can in nowise advantageously act upon the mucous membrane of the eye. Such a combination is worthy of the period which gave rise to it. It belongs to that epoch in therapeutics in which practitioners were persuaded that all agents capable of modifying the living economy were endowed with absolute curative properties, having nothing in common with the action they exercised on our organs, an action which they considered as generally hurtful and never useful. A fundamental error which time and experience have happily done justice to!—*Mialhe, l'Union Medicale,* No. 40.

4. *Sedative Cataplasm in Puerperal Arthritis.*—The following is the means of treatment adopted by M. Trousseau in that special form of articular phlegmasia which is frequently observed during the puerperal state at the Hôpital Necker—a means whence he declares he has derived the greatest advantage. It consists in the application of a cataplasm formed as follows:—As much bread as is required is boiled in camphorated brandy, and upon the cataplasm so formed a layer of camphor, consisting of about two or three drachms, is powdered over the poultice, and the whole moistened with a solution of the like quantity of extract of belladonna. It is to remain on for five or six days, and then to be renewed. Generally, on the very first night, the pain lessens, and after some days completely disappears. The resolution of the swelling is hastened, though much less rapidly.—*Bulletin de Therapeutique,* Feb.

5. *Disguise of the Bitterness of Medicines by means of Coffee.*—Medical men are perhaps scarcely sufficiently alive to the desirableness of masking the nauseousness of the abominable compounds they are forced to meddle with. It is not always desirable to do so, medicines of the anti-spasmodic and anti-hysteric class owing a proportion of their efficacy to their nastiness; while, again, it certainly is questionable whether the bitter taste of many medicines can be removed without impairing the value of the principle upon which this depends. However this may be, the statement of a M. *De Vouves,* a medical student at Paris, that the *bitter taste of Quinine may be completely masked by Coffee,* has excited considerable attention. M. *Dorvault,* Pharmacien, in a communication to the Union Medicale, furnishes the following formula, as, after repeated trials, being found to be the best for securing this object. Take of ground fresh-roasted coffee 10 parts, boiling water 100 parts. Treat it by displacement, strain and add Sulphate of Quinine 1 part, Sugar 15 parts—these two last having been previously well mixed together. The mixture must be well shaken when administered. For children milk may be added. He sums up his paper with these conclusions. 1. A solution of coffee annihilates completely, instantaneously, within wide limits, the bitterness of Quinine. 2. The disappearance of this taste is due in part to the transformation of the dissolved portion of the salt into a sort of tannate, and in part to other principles of the coffee. 3. Of all tanniferous substances coffee is most apt for this effect. 4. The therapeutical action of the medicine does not seem to be diminished.—*L'Union Medicale,* No. 32.

In another number of the same Journal, a M. *Combes,* a student in pharmacy, communicates the successful issue of a long series of trials upon the power *coffee possesses in masking the bitter taste of sulphate of magnesia*—a taste far more nauseous to most persons than is that of quinine. The following is his formula for an ordinary dose of about an ounce of the salt.

| | |
|---|---|
| Sulphate of Magnesia .. | 30 parts. |
| Ground Coffee.. .. . | 10 „ |
| Water .. .. .. | 700 or 800 „ |

Boil them briskly together for two minutes in an untinned vessel. Remove from the fire, and having allowed the mixture to infuse for a few minutes, strain it. Sugar it and drink it hot or cold, according to taste. To ensure the effect, the coffee must be boiled with the salt as directed above; adding the latter to it afterwards or to an infusion does not suffice. If the quantity of the sulphate be much increased, and it is yet desired not to add more coffee than the above, that will suffice if, while the fluid is boiling, a grain or two of tannin be added. —*L'Union Medicale,* No. 95.

The *Bulletin de Therapeutique* signalizes another use of *coffee* in *disguising the taste of Purgatives for Children;* MM. Guersant and Blache frequently employing it for this purpose. A weak decoction of coffee is made, to which some milk and sugar are added, care having been taken while boiling the coffee, to put in a *few follicles of senna.* If it is given to the children with a little bread, they will generally take it with avidity. This medicine generally acts freely upon children, and thus administered does not induce the violent griping it sometimes does in the adult. (As a matter of taste, we think the senna tea and prunes of our grandmothers is a more delicious preparation.)

6. *The Employment of Tonics in Acute Disease.*—M. Legroux, in a recent lecture at the Hôpital Beaujon, adverted to his successful treatment of some examples of acute disease by means of Tonics. He observed that, "in order properly to avail ourselves of the indications furnished by diseases, we should take into account their *foundation (fond)* and *form;* their *mode* and their *nature.* The *form* is the disease itself, which offers its general indications; the *foundation* is the idiosyncracy of the individual, which may modify and completely change the indication. The *mode* indicates the description of morbid process, but nowise its etiological nature, which in its turn furnishes new indications.

"It is especially in relation to *tonics* that the indications and contra-indications springing up between the relations or opposition which exist between the *foundation* and the *form* should be studied. And first, it is important to distinguish *true* from *apparent adynamia.* Prostration of strength with a good coloration of the skin, and sometimes with cyanic congestion of the face, occurring in a well-fed subject, having a small sub-inflammable pulse, but with a notable and persistent elevation of temperature, do not represent *true* adynamia. But if the patient is enfeebled by age, bad regimen, prior diseases, spontaneous or provoked deperditions; if he yields under an attempted antiphlogistic treatment; if the skin is cold; if the pulse in spite of its frequency is small and 'subinflammable,' if there are cold sweats, and on the assuming the erect posture a tendency to syncope, then the adynamia is real. In that case, whether the disease be a phlegmasia internal or external, an erysipelas, a pneumonia, or what not, we must abandon the form for the fundamental; and broths, wine, or quinine sometimes in such cases perform wonders. The tonic medication becomes antiphlogistic. It is especially indicated in persons habituated to the immoderate use of spirituous drinks, and whose idiosyncracy ill accommodates itself to active antiphlogistic measures.

The action of tonics under these circumstances may be easily understood if it is remembered that the capillary circulation in inflammations requires a certain degree of activity to effect the resolution of engorgements; and that a stimulant of a different character to that which has induced the congestion, and capable even itself of inducing the like in healthy tissues, will cause the disappearance of this. At the surface of the body we frequently find ourselves obliged to animate by topical applications languid inflammations; while the internal therapeutics opposed to the majority of the chronic inflammations of the lymphatic system are composed of stimulating medicinal substances.

One word as to rheumatism in particular. Suppose we have to do with a patient enfeebled by the duration of the disease, or by great deperditions. He may be pale, anæmic, wasted, tortured by cruel sufferings, his limbs rendered stiff by prolonged congestions, admitting of no movements, and his digestive organs being the seat of more or less acute pains, the stomach seeming to revolt at the slightest aliment. In these cases, again, the foundation is to be attended to before the form. We must restore the constitutional powers by ferruginous preparations and by substantial food, which will be perfectly well tolerated by stomachs that reject all insipid or non-azotized substances.

After adverting to the use of tonics in typhoid fever, Dr. Legroux concludes: " It is one of the most serious errors to "*dichotomize*" medical substances according to their properties; for their action is almost always complex. Stimulant and tonic at a slight or moderate dose, they may become great debilitants, or fatally hyposthenisant in larger doses. *Wine* is certainly the most powerful hyposthenisant we have; but in large quantities it debilitates and kills. In therapeutical doses *arsenic* is a tonic; but kills by hyposthenisation in a larger one. *Quinine* renders us high services as a tonic, but it is a hyposthenisant in large doses. The *tonic* medication then, cannot be regarded as simple in its effects. If it raises the fallen strength of a patient, so, on the other hand, may it moderate the febrile element, which is not incompatible with true adynamia.—*L'Union Medicale,* No. 86.

---

### M. Cazenave on Papular Diseases of the Skin.

The skin is a compound of very distinct organs, of the apparatus of different functions, some being entirely independent of each other, and others more or less connected in their physiological actions. Hence we might naturally be led to expect that each of these organs or apparatus would have its special lesion, giving rise to a special train of symptoms. The determination of *such elementary lesion* is by no means a matter of mere curiosity. The knowledge of the *seat* may lead, through the conditions of physiological pathology, and especially by the sympathies it discloses, to the demonstration of its *cause,* the appreciation of its *nature,* and the choice of its *treatment.*

If there is a form of disease of the skin in which the conditions of seat are in harmony with pathological observation, in which we can render an exact account of the phenomena observed by the anatomical composition of the affected tissue, it is beyond all doubt the *papular form.* Two very distinct characteristics distinguish eruptions of this class: the presence of small, full, solid, projecting elevations; and a more or less severe, but constant pruritus. The elevation is nothing else than the anormal development of the papillæ itself; a development which is easily appreciable from that permanent condition of "goose-skin" observed at the inner portion of the limbs of some persons, to the large papulæ of prurigo. If we bear in mind the nervous and vascular conformation of the papillæ we easily explain the various phenomena. Of these the *pruritus* is the dominant one, and it is a purely *nervous symptom.* In most cases the papillæ preserve the colour of the skin, and the vascular net-work takes no other part in the disease than as a slight congestion consequent on the hypertrophy of the papilla. In some forms indeed, as *prurigo vulvæ,* &c. the minutest examination fails to detect any enlargement whatever. Under other circumstances, the papillæ put on all the characters of a true *inflammation*; and in others the nervous and vascular elements may be found conjoined, as in *lichen agrius.* When the papular disease becomes chronic, and the papules become hard, dry and parchment-like—sometimes preserving a remarkable size, and no less remarkable thickness—the *epidermic* element is especially involved.

In examining into the intimate nature of these diseases, we remark their great affinity to other *nervous* diseases, with some of which they sometimes alternate. It is usually in women and sensitive individuals, in persons of a fine delicate skin, and at the periods of life when the nervous system is in highest activity, that these diseases are most frequent; and it is rare not to find them preceded or accompanied by other nervous disturbances, such as gastralgia, neuralgia, or uterine disorders. Moreover, the accessions of pruritus sometimes assume a periodicity only met with in the neuroses.

The papular eruptions present themselves under two distinct forms. In the one the papilla is often notably developed, but without any true inflammation; the nervous element and the epidermis of the papilla being alone interested. This is *prurigo*, an affection characterised by more or less intense pruritus, and by papulæ of different size, without change of colour of the skin, isolated, distinct, and accidentally tipped with little black crusts of coagulated blood; and when the disease has existed for a long time, by great thickening and condensation of the altered structure of the skin. In the other there may be less swelling than in the former, but there are also redness, and new products of inflammation, the whole elements of the papillæ being now affected. This is *lichen*, a disease often characterised by intense pruritus, accompanied by solid, full, papulæ, generally very small, agglomerated, and sometimes confluent. They are generally red, and may go on to ulceration, the secretion of a sero-purulent fluid, scales and even crusts, and later to a thickening of the skin, which takes on a peculiar yellowish tinge. In point of treatment the distinction of the two forms is not so important as might be supposed. All depends upon the morbid predominance of this or that element. If the exaggeration of the sensibility is very predominant, anti-spasmodics are given, hygienic precautions adhered to, and especially a non-stimulant regimen ordered. M. Cazenave has tried a great quantity of substances but has found most benefit from the use of *Extract of Aconite* (1 to 2 grs. per diem), which, together with a few baths, has frequently enabled him to triumph over even general papular eruptions. *Topical applications* are seldom required, save when the epidermis has become hypertrophied. In the *acute stage* of papular eruption, which is represented by the varieties of *lichen*, the application of topics, and especially of ointments, is generally hurtful. M. Cazenave has seen, under the use of the simplest ointments, simple lichen become violently inflamed and go on to ulcers. In the *chronic* state, however, constituted by *prurigo*, ointments are usually well borne, and advantageously modify the disease in most cases, although they do not procure its cessation. When there is distressing *pruritus* topical applications are essential, those of a narcotic character being often useful in irritable subjects. In other cases tar-water, alkaline lotions (subcarb. potass 8 to 12 parts to 500 of water), or sulphuretted lotions (sulphuret of potass 4 p. wtaer 300), are most so.

In chronic *eczema*, and especially in *pruritus without eruption*, M. Cazenave has found the formula recommended by Biett (Cyanuret of Potass gr. x., Emulsion of Bitter Almonds, ℥vj.), frequently very useful. In spite of all means, the itching often remains with provoking obstinacy. It may be allayed sometimes by camphor (Camphor 30 p., Lettuce water 500) when it is very intense. But of all topics *mercurial* lotions usually best counteract this. In the dreadful suffering arising from *pruritus vulvæ*, a little of the following may be employed. Bichlor. Hydr. gr. iv., Spt. Menthe ℥ss., Aq. dest. ℥vj.—*L'Union Medicale*, No. 98.

---

### Treatment of Obstinate Hiccough by Prolonged Compression of the Epigastrium.

Dr. Boyer relates three cases of prolonged and alarming hiccough, which, having resisted all the usual means employed for its relief, were relieved by the application of pressure, a practice first suggested by Bordeu and since revived by M. Rostan. A large pad is laid on the epigastrium, and bound forcibly on by means of a towel or bandage. It generally causes instant relief, but if discontinued too soon the hiccough returns. It is usually necessary to wear it for twenty-four hours, before it can be safely removed.—*Revue Medico-Chirurgicale*, July.

### Account of a Physical Sign of Pneumonia of the Apex of the Lung. By Wm. Boling, M.D.

The experience of Dr. Boling is confirmatory of the opinion that, when pneumonia commences at the apex of the lung it is especially fatal; and his object in the present communication is to indicate a physical sign which may lead to its earlier diagnosis. "This is a fine mucous or crepitant rhonchus, *seemingly seated in the larynx,* loud enough to be heard distinctly at the distance of two or three feet from the patient, and so *persistent,* that it is not removeable, or but momentarily, by any effort to expectorate which the patient may make, while at the same time there are present none of the signs of bronchitis or laryngitis." Though seeming to the by-stander to arise from mucus in the larynx, the indifference manifested by the patient proves this is not the case; and on applying the stethoscope just above or below the clavicles it will be found to proceed from the apex of the inflamed lung. "It would seem that the sound there produced in the pulmonary vesicles must be conveyed by the larger bronchial ramifications, numerous and superficial at this point, to the larynx, where, in consequence of the thinness of the tube, or rather the thinness of its covering, and its proximity to the surface, the deceptive impression of its production in this organ, from the presence of a small quantity of viscid mucus, is created. It is the indifference of the patient to the presence of the sound, but still more especially, its *persistence,* which constitutes its peculiar and distinctive feature, and upon which its value as an evidence of pneumonia commencing in the apex of the lung depends."—*American Journal Med. Sciences,* July 1847.

---

### New Modes of effecting Union of Wounds.

M. *Amussat* has of late procured the union of large wounds by the first intention by means of the following suture. He passes several very fine steel sewing needles through the cutaneous edges of the wound, and having twisted a waxen thread around them, breaks off their extremities by means of a forceps, and leaves them to fall out of themselves, which they do in a few days, just as ligatures of vessels are allowed to do.—*Gazette des Hopitaux,* No. 69.

M. *Baudens* employs the following means for bringing together the edges of wounds. Speaking of that resulting from an amputation for example, he directs a circular bandage to be placed above the stump, and two strong pins fixed into this, one before and one behind, in such a manner as to leave their heads and points exposed. A double point of support is thus got, around which strong cotton threads are passed; these are crossed over each other towards the face of the stump in such a manner as to draw the integuments together with any desired force, after the manner of an uniting bandage, terminating with a figure of eight, just as in the operation for hare-lip.—*Comptes Rendus,* T. 24, p. 1018.

---

### Chronic Cutaneous Eruptions.

M. Cazenave recommends the following formula as of excellent service in chronic dartrous eruptions, as impetigo, eczema, lupus, and all diseases of the skin allied to the lymphatic and scrofulous constitutions.

Crystallized Chloride of Lime . . . . 15 parts.
Distilled Water . . . . . . . . . . . . 500

A tablespoonful three times daily in some bitter infusion.

